# Transcriptional and imaging-genetic association of cortical interneurons, brain function, and schizophrenia risk

**DOI:** 10.1038/s41467-020-16710-x

**Published:** 2020-06-08

**Authors:** Kevin M. Anderson, Meghan A. Collins, Rowena Chin, Tian Ge, Monica D. Rosenberg, Avram J. Holmes

**Affiliations:** 1 Department of Psychology, Yale University, New Haven, CT 06520 USA; 2 Psychiatric and Neurodevelopmental Genetics Unit, Center for Genomic Medicine, Massachusetts General Hospital, Boston, MA 02114 USA; 3 Department of Psychiatry, Massachusetts General Hospital, Harvard Medical School, Boston, MA 02114 USA; 4 Department of Psychology, University of Chicago, Chicago, IL 60637 USA; 5 Department of Psychiatry, Yale University, New Haven, CT 06520 USA

**Keywords:** Gene expression, Cognitive neuroscience, Schizophrenia

## Abstract

Inhibitory interneurons orchestrate information flow across the cortex and are implicated in psychiatric illness. Although interneuron classes have unique functional properties and spatial distributions, the influence of interneuron subtypes on brain function, cortical specialization, and illness risk remains elusive. Here, we demonstrate stereotyped negative correlation of somatostatin and parvalbumin transcripts within human and non-human primates. Cortical distributions of somatostatin and parvalbumin cell gene markers are strongly coupled to regional differences in functional MRI variability. In the general population (*n* = 9,713), parvalbumin-linked genes account for an enriched proportion of heritable variance in in-vivo functional MRI signal amplitude. Single-marker and polygenic cell deconvolution establish that this relationship is spatially dependent, following the topography of parvalbumin expression in post-mortem brain tissue. Finally, schizophrenia genetic risk is enriched among interneuron-linked genes and predicts cortical signal amplitude in parvalbumin-biased regions. These data indicate that the molecular-genetic basis of brain function is shaped by interneuron-related transcripts and may capture individual differences in schizophrenia risk.

## Abstract (web summary)

Interneuron subtypes have distinct properties and spatial distributions. Here, the authors show that the molecular-genetic basis of cortical resting-state brain function is shaped by distributions of interneuron-related transcripts and may capture individual differences in schizophrenia risk.

## Introduction

Ramón y Cajal theorized that the functional diversity of the human brain arises, in part, from the vast assortment of neurons that pattern cortex^1^. Inhibitory interneurons are the most varied neuronal class^2^, exhibiting divergent morphological and physiological properties while coordinating information flow across the brain’s collective set of connections (functional connectome)^3^. Converging animal and human work provides evidence for the role of interneurons in healthy brain function as well as their dysregulation in psychiatric illnesses, including schizophrenia^4^ and major depressive disorder^5^. The development of dense spatial transcriptional atlases now enables the study of cellular and molecular associates of functional brain networks. Early work in this area identifies genes encoding ion channels^6^ and those enriched in supragranular layers of cortex^7^ as correlates of large-scale network organization. Other research indicates that cortical resting-state signal fluctuations follow the expression of neuron-enriched genes^8^. However, the transcriptional correlates of brain function have rarely been validated in-vivo^6^ and little is known about how the spatial distribution of specific interneuron subtypes shapes cortical function and associated risk for psychiatric illness in humans.

The spatial distribution of interneuron subtypes is theorized to contribute to regional specialization of cortex, partly by altering the relative balance of excitation and inhibition for a given cortical area^9–11^. Interneurons comprise 20–30% of cortical neurons^12^ and form stereotyped connections with excitatory projection neurons^2,3^. The majority of interneurons express one of a limited set of genetic markers: somatostatin (*SST*), parvalbumin (*PVALB*), and vasoactive-intestinal peptide (*VIP;* a subset of *HTR3A* interneurons)^2^. Each subtype possesses unique synaptic and functional characteristics, leading to the hypothesis that the ratio of interneuron classes underpins local differences in neural activity^11,13^. For example, SST interneurons preferentially target dendrites of cortical projection neurons to regulate their input, whereas PVALB interneurons synapse on perisomatic regions to regulate output^2^. Consequently, increased relative density of SST interneurons may facilitate filtering of noisy or task-irrelevant cortical signals and promote recurrent excitation required for higher-order cognition^11,14^. Conversely, relative increases in PVALB may produce stronger feedback inhibition on excitatory neurons, leading to shorter activation timescales suited for processing constantly changing sensorimotor stimuli^11,15^. Previous work documents cortical gradients of gene transcription that mirror the hierarchical organization of timescales from fast (unimodal cortex) to slow (multimodal cortex)^8,10,16,17^. Further, SST and PVALB interneuron markers are differentially expressed within distributed limbic and somato-motor cortico-striatal networks, respectively^9^. These observations suggest that spatial distributions of interneuron subtypes could underlie regional signaling differences across the cortical sheet, as indexed by blood oxygenation level-dependent (BOLD) functional magnetic resonance imaging (fMRI). However, the psychiatric and functional consequences of spatially variable SST- and PVALB-related transcription in cortex have yet to be fully characterized.

Establishing the principles by which cellular diversity influences brain function is a long-standing challenge in neuroscience and could reveal biological mechanisms of individual variability of the human brain. Consistent with this aim, cross-species evidence indicates the importance of PVALB interneurons for fMRI measures of brain function^18^. PVALB interneurons orchestrate gamma-band oscillations (30–80 Hz^19^), a frequency range that is tightly coupled to spontaneous BOLD fluctuations^20^. Experimental optogenetic stimulation of PVALB interneurons in rodents drives gamma-band rhythms, impacting information processing through the synchronization of excitatory neurons^19^. In psychiatric illness, decreased PVALB-mediated inhibition may be a core locus of disruption in schizophrenia, leading to altered gamma-band signals and working memory deficits that are a hallmark of the disorder^21^. However, a direct link between PVALB-related genetic variation and human brain activity has yet to be established. Linking cortical interneurons to individual differences in human brain function would yield deep biological insight into the hemodynamic BOLD signal, providing an engine for the discovery of functional connectome-linked genes and associated risk for illness onset.

Here, we bridge genetic, transcriptional, and neuroimaging data to advance three lines of inquiry linking interneurons to human brain function. First, we describe the organization of *SST* and *PVALB* expression in human and non-human primates, demonstrating a robust pattern of anti-correlation across cortex and subcortex. We perform single-cell polygenic deconvolution^22^ of bulk cortical tissue data to infer spatial distributions of inhibitory, excitatory, and non-neuronal cells across cortex, providing converging evidence with single-marker analyses. Second, we establish that the relative density of *SST* and *PVALB* tracks regional differences in cortical brain activity. In a large sample (*N* = 9713)^23^, genetic variation among *PVALB*-correlated genes explained an enriched proportion of heritable variance in resting-state signal amplitude (RSFA), in a manner that mirrors the spatial expression of *PVALB* measured in independent post-mortem brain tissue. These discoveries suggest that the molecular-genetic basis of cortical function is spatially nonuniform and that genes linked to PVALB interneurons explain heritable aspects of the BOLD signal. Third, we link PVALB interneurons and psychotic illness, demonstrating that genetic risk for schizophrenia is enriched among interneuron-linked genes and predicts reduced resting-state signal amplitude in a spatially heterogeneous manner that follows *PVALB* expression. These data help address a deep-rooted challenge in neuroscience to understand how cytoarchitecture shapes human brain function and related vulnerability for psychiatric illness.

## Results

### Anti-correlation of SST and PVALB interneuron markers across cortex

The properties of interneuron subtypes emerge early in development and are partly determined by their spatial origin in the embryonic ganglionic eminence^24^. SST and PVALB interneurons originate in the medial ganglionic eminence (MGE) along negatively correlated spatial gradients^25^. That is, PVALB- and SST-destined neurons differentially cluster within the dorsal and ventral MGE, respectively^26^. Evidence in humans^9,10^ and rodents^11,17^ suggests that SST and PVALB transcripts maintain a negative spatial correlation in adulthood, indicating that embryonic organization may constitute a “proto-map” of mature cortex. Although prior research suggests that SST and PVALB markers are differentially expressed across cortex^9,10^, the current work directly establishes transcriptional anti-correlation between these two cell types across multiple techniques, human datasets, non-human primate data, and human neurodevelopment. This comprehensive profiling of SST and PVALB interneuron expression is necessary for deep profiling of their relationship to in-vivo brain function and subsequent schizophrenia risk through statistical genetic approaches. The functional consequences of a negative spatial SST to PVALB relationship are not well understood, but the presence of replicable and evolutionarily conserved expression patterns may indicate the importance of interneuron gradients.

**Fig. 1:**
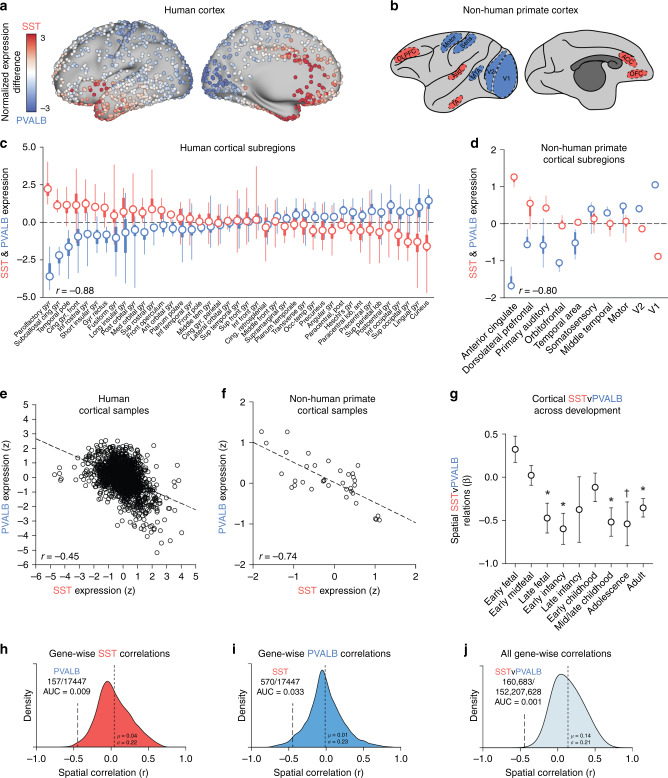
Cortical expression of *SST* and *PVALB* are negatively correlated across species and developmental stages. **a** AHBA tissue samples mapped to the human cortical surface, and **b** an illustration of non-human primate tissue sample locations, colored by relative expression of *SST* (red) and *PVALB* (blue). Normalized expression difference reflects the sample-wise subtraction of *z*-transformed *PVALB* from *SST*. Relative *SST*–*PVALB* expression among anatomically defined groups from the **c** AHBA (*n* = 6 donors; *n* = 1683 samples; *n* = 41 regions) and **d** NIH Blueprint Non-Human Primate Atlas (*n* = 4 donors; *n* = 182 samples; *n* = 10 regions); circles = median, thick lines = interquartile range, thin lines = min and max values. **e** Sample-wise negative correlation of *SST* and *PVALB* in human cortex (*r* = −0.45, *p* < 2.2e−16; *r*_s_ = −0.40, *p* < 2.2e−16) and **f** non-human primates (*r*(34) = −0.74, *p* = 2.2e−7; *r*_s_ = −0.60, *p* = 0.0001). **g** Correlation of cortical *SST* and *PVALB* across nine developmental stages using data from the Brainspan Atlas of the Developing Human Brain (*n* = 42 donors; *n* = 362 samples; *n* = 9 developmental stages). **h**–**j** The *SST* to *PVALB* correlation is at the left tail of the distribution of all gene-wise correlations to *SST* (AUC = 0.009) and to *PVALB* (AUC = 0.033), as well as all possible two-gene spatial correlations (AUC = 0.001). *p ≤ 0.05, uncorrected; ^†^*p* ≤ 0.10; error bars = standard error.

To characterize interneuron marker topography across human and non-human primate cortex, we analyzed gene expression data from the Allen Human Brain Atlas (AHBA)^27^ and NIH Blueprint Non-Human Primate (NHP) Atlas^28^. AHBA cortical samples from the left (*n* = 1265) and right (*n* = 418) hemispheres were analyzed. Microarrays do not provide absolute estimates of gene transcription, but can measure within-probe differences across samples. *SST* and *PVALB* expression were thus *z*-transformed across cortical samples, and subtracted (i.e., *SST*–*PVALB*) to reveal relative expression differences (Fig. 1a). Extending prior evidence^9,10^, *SST* and *PVALB* were anti-correlated across AHBA cortical samples (Fig. 1e) for both parametric (*r*(1681) = −0.45, *p* < 2.2e−16) and non-parametric tests (Spearman’s rho (*r*_s_) = −0.40, *p* < 2.2e−16). Relative to all possible gene-wise correlations, the *SST* to *PVALB* correlation was among the most negative (Fig. 1h, i; AUC_sst_ = 0.009, AUC_pvalb_ = 0.033). Further, the *SST* and *PVALB* relationship was among the most negative relative to all possible (*n* = 152,207,628) two-gene pairings (Fig. 1j; AUC = 0.001). *SST* and *PVALB* distributions were organized along an anterior to posterior gradient, with greatest relative *SST* in orbitofrontal and medial prefrontal cortex, anterior insula, and the temporal lobe (Fig. 1a, c and Supplementary Fig. 1). By contrast, relative *PVALB* expression was greatest within unimodal somato-motor, parietal, and visual cortices (Fig. 1a, c and Supplementary Fig. 1). Histologically defined anatomical categories were used to characterize regional differences of interneuron marker expression, reflected in Fig. 1c showing negatively correlated *SST* and *PVALB* median across cortical subregions (*r*(39) = −0.88, *p* = 2.4e−14).

The negative spatial relationship between *SST* and *PVALB* was evolutionarily conserved in non-human macaque primates (Fig. 1f; *r*(34) = −0.74, *p* = 2.2e−7; *r*_s_ = −0.60, *p* = 0.0001), suggesting that interneuron marker gradients may reflect a core organizational feature of primate cortex. These data complement evidence for similar negatively correlated SST and PVALB gradients in rodents^11,17^. Given that SST and PVALB interneurons originate along stereotyped, anti-correlated gradients in the MGE^26^, we analyzed RNAseq data from the Brainspan Atlas of the Developing Human Brain to test whether the emergence of *SST* to *PVALB* negative correlations coincides with major waves of interneuron colonization, approximately 10–25 post-conception weeks (pcw)^29,30^. The negative correlation between *SST* and *PVALB* was absent in early-fetal (8–12 pcw; *β* = 0.32, *p* = 0.039) and early-midfetal (13–21 pcw; *β* = 0.02, *p* = 0.85) stages. Consistent with the hypothesis that mature interneuron distributions result from developmentally programmed migration patterns, we observed significant negative correlations between *SST* and *PVALB* emerge during late-fetal (24–37 pcw; *β* = −0.47, *p* = 0.012), early-infancy (4 months; *β* = −0.60, *p* = 0.0033), mid-late childhood (8–11 years; *β* = −0.52, *p* = 0.0038), and adult (18–40 years; *β* = −0.35, *p* = 0.0014) periods, as well as at a trend-level in adolescence (13–15 years; *β* = −0.54, *p* = 0.057). We did not observe a relationship in late infancy (10 months; *b* = −0.37, *p* = 0.36) or early childhood (1–4 years; *b* = −0.11, *p* = 0.48). These data provide developmental context as well as an external replication of the *SST*–*PVALB* cortical expression pattern observed in the AHBA (adult human) and NHP Atlas (adult macaque) samples.

### Polygenic deconvolution of cell types across cortex

*SST* and *PVALB* are reliable genetic markers of their respective interneuron subtypes^2^, however single-cell transcriptomics show that cell classes possess polygenic signatures of expression^31^. To capture the molecular complexity of cell identity and quantify cell-level associates of brain function, we conducted polygenic cellular deconvolution of bulk AHBA expression data using CIBERSORTx (https://cibersortx.stanford.edu/)^22^. This method leverages transcriptomic “signatures” of cellular identity to estimate the relative abundance of cell types in bulk tissue data. Single-nucleus droplet-based sequencing (snDrop-seq) data from Lake and colleagues^31^ was used, providing cell-level expression data in dorsal frontal cortex (BA6/BA10; 10,319 cells) and visual cortex (BA17; 19,368 cells). Collinearity among transcriptionally similar cell types was reduced by using 18 superordinate cell identities defined by Lake and colleagues^31^. Cell signatures were created with CIBERSORTx (Supplementary Fig. 5) and cell abundance, expressed as a fraction, was estimated for each cortical tissue sample from AHBA donors (see Supplementary Data). AHBA tissue samples were projected to the cortical surface, allowing for vertex-level localization on a group-atlas (fsLR32k; see “Methods”). Fractional cell abundances were averaged for each of the 400 bi-hemispheric parcels from the atlas of Schaefer and colleagues^32^ (Supplementary Figs. 6–8).

**Fig. 2:**
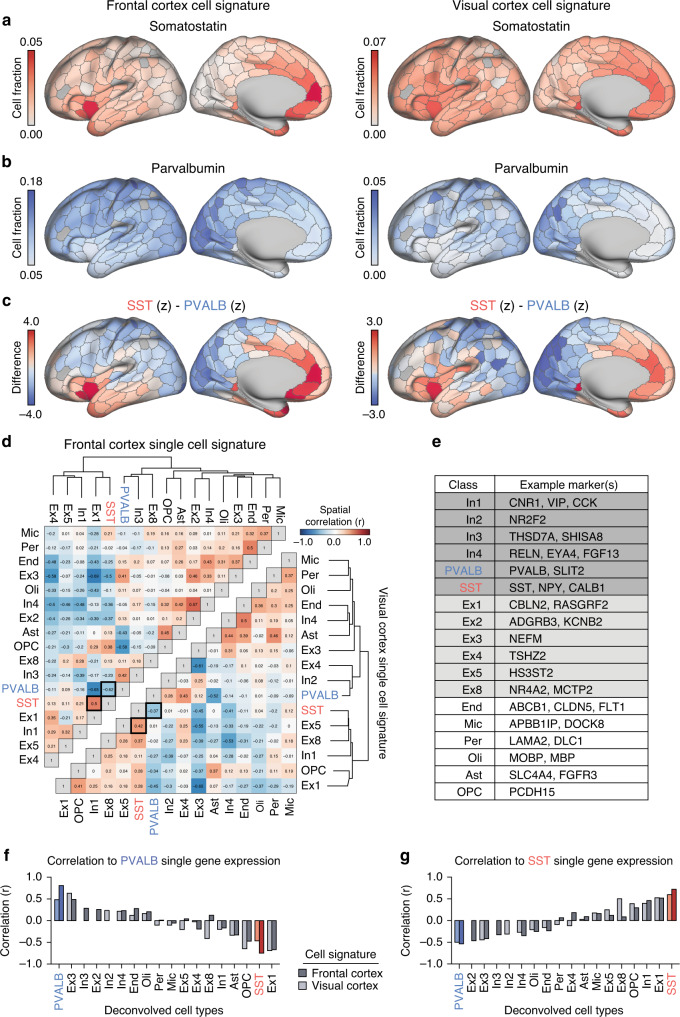
Deconvolved cell type distributions are consistent with SST and PVALB single marker expression maps. Using CibersortX^22^, frontal and visual cortex snDrop-Seq data from Lake and colleagues^31^ were used to deconvolve cell type fractions from bulk AHBA microarray expression data. Deconvolved cell fractions of **a** somatostatin and **b** parvalbumin interneurons across cortex using single-cell data from frontal (left) and visual (right) cortex. **c** Somatostatin and parvalbumin cell fraction maps were *z*-transformed and subtracted (SST–PVALB) to illustrate the relative density of each subtype across cortex. **d** Spatial correlations of each deconvolved cell type across cortex using frontal (top-left triangle) and visual (bottom-right triangle) cell signatures. **e** Example marker genes for each cell class. **f** Deconvolved parvalbumin fractions across cortex were positively spatially correlated (Pearson’s) to single-gene PVALB expression (frontal cortex: *r*(337) = 0.81, *p* < 2.2e−16; visual cortex: *r*(337) = 0.48, *p* < 2.2e−16). **g** Deconvolved somatostatin fractions across cortex are positively spatially correlated (Pearson’s) to single-gene SST expression (frontal cortex: *r*(337) = 0.72, *p* < 2.2e−16*;* visual cortex: *r*(337) = 0.60, *p* < 2.2e−16).

We focus on imputed spatial distributions of SST and PVALB interneurons across cortex, estimated separately from visual and frontal cortex single-cell data (Fig. 2). Similar to single-marker analyses (Fig. 1a and Supplementary Fig. 1a), the inferred fractional abundance of SST interneurons was greatest in medial PFC, anterior insula, and temporal poles (Fig. 2a), which was highly consistent between frontal and visual cortex cell type derived maps (*r*(337) = 0.73, *p* < 2.2e−16). Conversely, PVALB fractional abundance was highest within visual, motor, and dorsal parietal cortex (Fig. 2b), which was also consistent across frontal and visual cortex derived estimates (*r*(337) = 0.76, *p* < 2.2e−16). Given the hypothesized importance of relative SST and PVALB abundance for cortical functional dynamics, Fig. 2c shows the spatial distribution of estimated SST- (red) and PVALB-dense (blue) areas of cortex. Figure 2d illustrates the spatial correlation of all deconvolved cell type fractions across cortex, recapitulating the negative spatial relationship between *SST* and *PVALB* single-gene markers (frontal cortex: *r*(337) = −0.62, *p* < 2.2e−16; visual cortex: *r*(337) = −0.37, *p* = 1.15e−12). Critically, cell fraction maps of PVALB and SST obtained through polygenic deconvolution were strongly correlated with the expression of their respective single-marker genes (Fig. 2f, g).

### SST and PVALB anti-correlation in subcortical territories

**Fig. 3:**
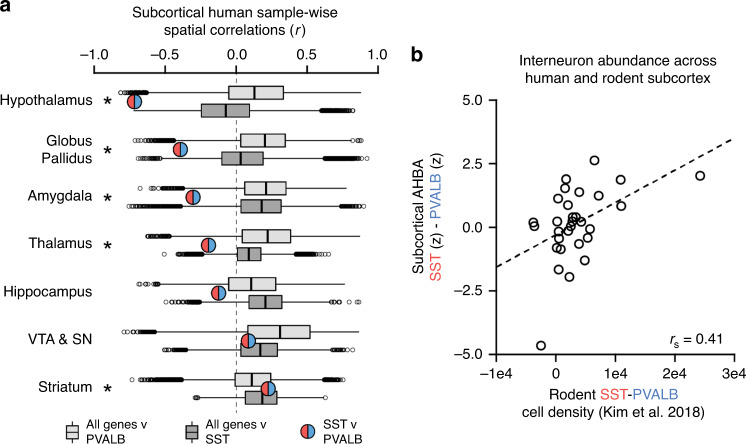
Subcortical *SST* and *PVALB* correlation and consistency in rodents. **a** The correlation between *SST* and *PVALB* was estimated using AHBA data for each of seven subcortical territories. Red/blue circles denote Pearson correlations between *SST* and *PVALB*. Light and dark gray boxplots show the distribution of spatial correlations of all other genes (*n* = 17,447) to *PVALB* and *SST*, respectively (center = median, box = Q1–Q3, whiskers = 1.5*IQR, circles = outliers). The strength of the *SST* to *PVALB* relationship is quantified relative to gene-wide reference distributions. *SST* was more negatively correlated to *PVALB* than what is expected by chance in the hypothalamus (*r*(100) = −0.72, *q* = 1.9e−16, AUC_sst_ = 0.002, AUC_pvalb_ = 0.01), globus pallidus (*r*(37) = −0.39, *q* = 0.011), amygdala (*r*(65) = −0.30, *q* = 0.019), and thalamus (*r*(173) = −0.19, *q* = 0.019), but not the hippocampus (*r*(156) = −0.12, *q* = 0.14), ventral tegmentum/substantia nigra (*r*(63) = 0.09, *q* = 0.50), and striatum (*r*(168) = 0.23, *q* = 0.011). **b** Relative expression of *SST* and *PVALB* in human subcortical areas when compared to ground truth cell densities in rodent homologs from Kim et al.^11^ (Spearman’s *r*_s_ = 0.41, *p* = 0.025).

We next examined whether the negative spatial relationship between *SST* and *PVALB* is also preserved across subcortex (see Supplementary Information for subcortical sample information). Although there is evidence for anti-correlated *SST* and *PVALB* interneuron gradients in striatum^9^ and hippocampus^33^, it remains unclear whether *SST* and *PVALB* inverse gradients are ubiquitous throughout the brain. Sample-wise expression was normalized and correlated separately for each of the seven areas: striatum, dorsal thalamus, hypothalamus, globus pallidus, amygdala, hippocampus, and combined substantia nigra/ventral tegmentum. The strength of the *SST* to *PVALB* correlation was benchmarked against all gene-wise correlations to *SST* and *PVALB* (Fig. 3a), and quantified as AUC of the distribution less than or equal to the *SST*–*PVALB* correlation. *SST* was significantly negatively correlated to *PVALB* in the hypothalamus (*r*(100) = −0.72, *q* = 1.9e−16, AUC_sst_ = 0.002, AUC_pvalb_ = 0.01), globus pallidus (*r*(37) = −0.39, *q* = 0.011, AUC_sst_ = 0.03, AUC_pvalb_ = 0.03), amygdala (*r*(65) = −0.30, *q* = 0.019, AUC_sst_ = 0.06, AUC_pvalb_ = 0.03), and thalamus (*r*(173) = −0.19, *q* = 0.019, AUC_sst_ = 0.04, AUC_pvalb_ = 0.07), but not the hippocampus (*r*(156) = −0.12, *q* = 0.14, AUC_sst_ = 0.06, AUC_pvalb_ = 0.21), ventral tegmentum/substantia nigra (*r*(63) = 0.09, *q* = 0.50, AUC_sst_ = 0.39, AUC_pvalb_ = 0.32), and striatum (*r*(168) = 0.23, *q* = 0.011, AUC_sst_ = 0.65, AUC_pvalb_ = 0.72).

These data identified subcortical territories with biased *SST* or *PVALB* expression in humans, which show striking correspondence to non-human animal literature^11^. Median *z*-transformed *SST* and *PVALB* was calculated for subdivisions of each subcortical region (Supplementary Fig. 9). Across 40 AHBA subcortical sub-regions, 31 were able to be mapped onto rodent homologs present in cell density (cells per mm^3^) data from Kim and colleagues^11^. The relative presence of human *SST* and *PVALB* across sub-regions was significantly positively correlated to ground truth cell density estimates in rodents (Fig. 3b; *r*_s_ = 0.41, *p* = 0.025). Results were consistent when Spearman-rank correlation was used to limit the influence of outliers, and when the ratio of SST/PVALB rodent cell densities was used rather than the difference (*r*_s_ = 0.49, *p* = 0.006).

In particular, the central nucleus (CeA) of the amygdala was enriched for SST in both rodents (SST_density_ = 24,342.60, PVALB_density_ = 119.42) and humans (SST_expr(z)_ = 1.27, PVALB_expr(z)_ = −0.76), paralleling observations in primates^34^. We also found greater relative SST in the ventral tegmental area (VTA) for both rodents (SST_density_ = 1953.98, PVALB_density_ = 426.10) and humans (SST_expr(z)_ = 1.12, PVALB_expr(z)_ = −0.42), but greater relative presence of PVALB in the substantia nigra pars reticulata (SNr) in humans (SST_expr(z)_ = 0.22, PVALB_expr(z)_ = 0.72) and rodents (SST_density_ = 680.27, PVALB_density_ = 4373.48). Supporting this VTA/SNr distinction, the VTA is connected to other *SST*-biased regions, including the nucleus accumbens (NAcc), anterior cingulate cortex, and mediodorsal thalamus^35^, whereas functional neuroimaging indicates a preferential coupling of the SNr to motor areas and sensorimotor striatum^36^. Building on the previous work^9^, the extent to which *SST* may be relatively increased within a distributed limbic cortico-striato-thalamic network is explored in Supplementary Fig. 10.

### Relative SST and PVALB covaries with resting-state signal amplitude

**Fig. 4:**
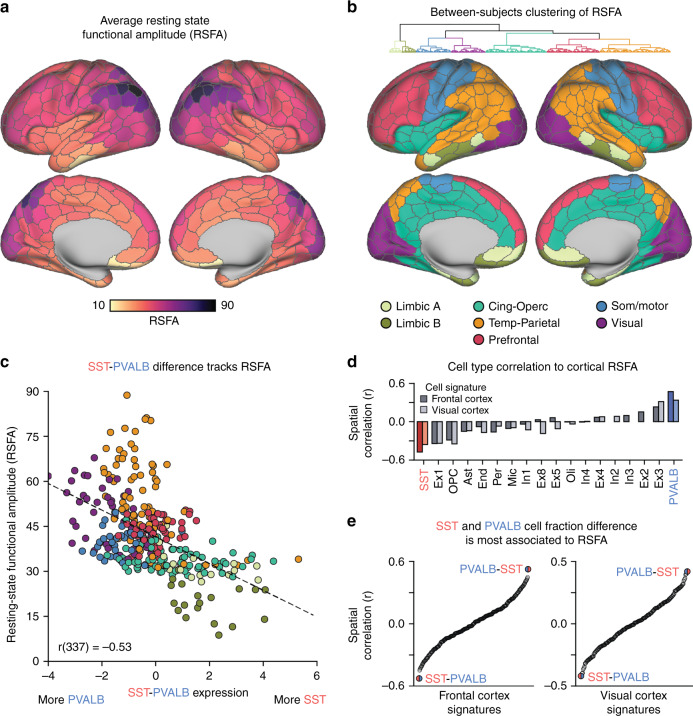
*SST*–*PVALB* difference tracks inter-regional variation in cortical brain function. **a** RSFA across each of the 400 Schaefer atlas parcels, averaged across 9713 UKB subjects. **b** Between-subject hierarchical clustering of residualized RSFA reveals 7-clusters cortical partitions with similar amplitude signatures; Light beige = limbic A, dark beige = limbic B, teal = cingulo-opercular, orange = temporal-parietal, red = prefrontal, blue = somato/motor, and purple = visual. **c** Relative presence of SST–PVALB is negatively correlated (Pearson’s) with cortical RSFA (*r*(337) = −0.53, *p* < 2.2e−16). **d** Across all deconvolved cell types, RSFA is most negatively spatially correlated (Pearson’s) to SST cell fractions (frontal cortex signature: *r*(337) = −0.48, *p* < 2.2e−16; visual cortex signature: *r*(337) = −0.36, *p* = 1.2e−11) and most positively correlated to PVALB cell fraction (frontal cortex signature: *r*(337) = 0.47, *p* < 2.2e−16; visual cortex signature: *r*(337) = 0.34, *p* = 1.7e−10). **e** Across frontal cortex (*n* = 272) and visual cortex (*n* = 240) cell type pairs, the relative difference of SST and PVALB cell fractions is most spatially associated with cortical RSFA.

**Fig. 5:**
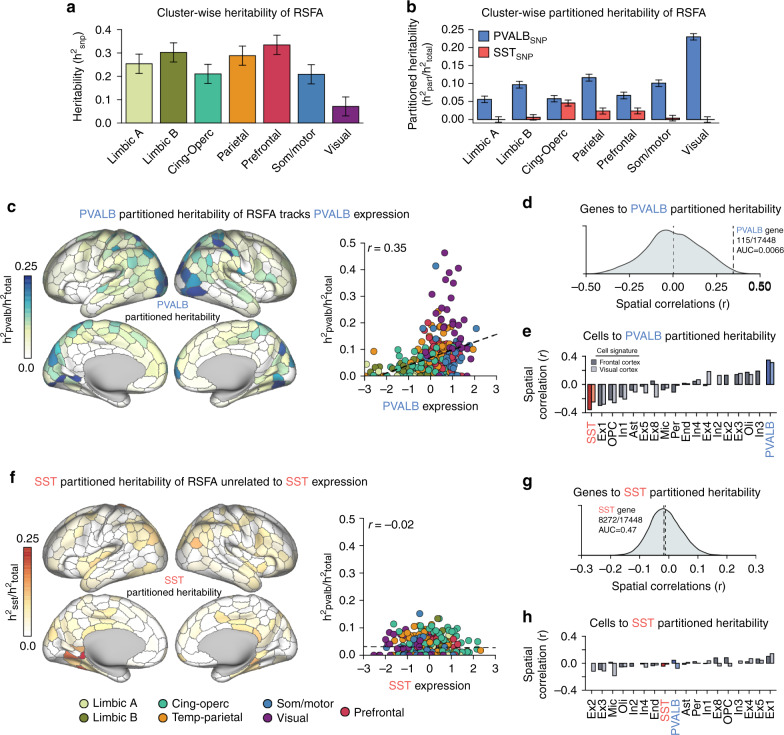
*PVALB*-linked genetic variation explains patterns of heritable brain function. **a** RSFA was significantly heritable across the seven empirically defined spatial clusters (*n* = 9713 UKB subjects). **b** Partitioned heritability analyses reveal that the 500-gene PVALB_SNP_ set accounted for a significant proportion of heritable variance in all seven clusters (*n* = 9713 UKB subjects). **c** Parcel-wise PVALB_SNP_ partitioned heritability of RSFA (left panel) tracks the *PVALB* single-gene expression across cortex (Pearson’s *r*(326) = 0.35, *p* = 1.04e−10; *r*_s_ = 0.40, *p* = 2.2e−14). **d** Across all genes, *PVALB* was the 115th most correlated gene to PVALB_SNP_ partitioned heritability (AUC = 0.007). **e** Across all deconvolved cell types, inferred PVALB cell fraction was the most positively correlated to PVALB_SNP_ partitioned heritability (frontal cortex: Pearson’s *r*(329) = 0.35, *p* = 2.27e−11; *r*_s_ = 0.39, *p* = 2.20e−13; visual cortex: Pearson’s *r*(329) = 0.31, *p* = 6.67e−9; *r*_s_ = 0.34, *p* = 1.50e−10). **f** SST_SNP_ partitioned heritability was not associated with *SST* single-marker expression (Pearson’s *r*(326) = −0.02, *p* = 0.75), a null finding that was consistent when put in context of all genes (**g**) and inferred cell types (**h**). Error bars = standard error.

**Fig. 6:**
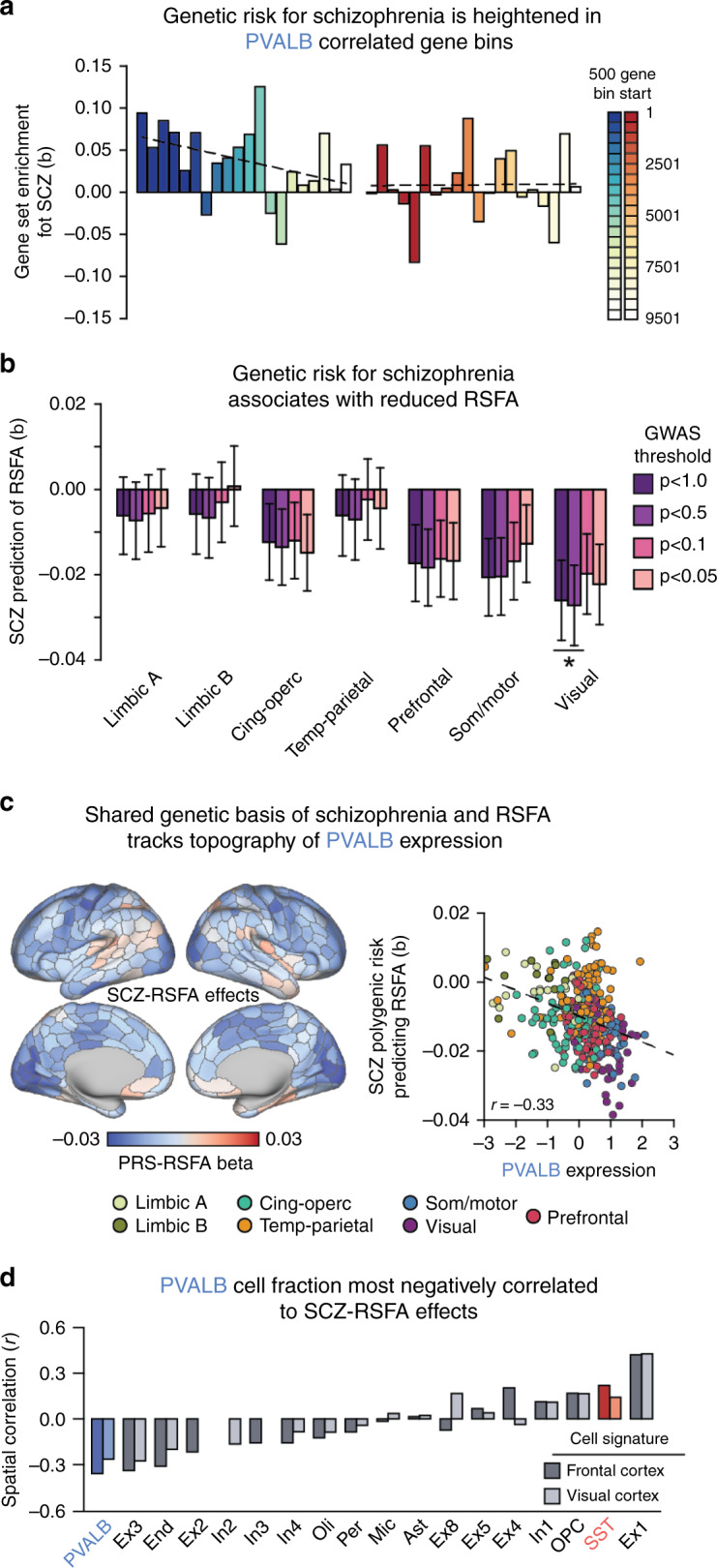
Schizophrenia polygenic risk predicts brain function and tracks *PVALB* expression. **a** Genes were rank-ordered by cortical spatial correlation to *SST* and *PVALB*, then divided into 500-gene bins. MAGMA competitive gene set analysis revealed enrichment of polygenic risk for schizophrenia in the top *PVALB* (*p* = 0.022), but not the top *SST* (*p* = 0.51) set. Enrichment decreased across ordered bins for *PVALB* (Spearman’s *r*_s_ = −0.48, *p* = 0.03) but not for *SST* (Spearman’s *r*_s_ = −0.001, *p* = 0.51). **b** Schizophrenia polygenic risk negatively predicts RSFA within the visual (*q*_1.0_ = 0.04) cluster, as well as somato/motor (*q*_1.0_ = 0.08) and prefrontal (*q*_1.0_ = 0.10) clusters at trend-levels (corrected for multiple-comparisons). **c** Parcel-wise prediction of RSFA by the schizophrenia PRS negatively correlated with cortical expression of *PVALB* (Pearson’s *r* = −0.33, *p* = 3.1e−10), which was also significant relative to all genes (PVALB = 145/17,448, AUC = 0.008). **d** Across all deconvolved cell type distributions, PVALB was the most negatively correlated to cortical SCZ-RSFA effects (frontal cortex: Pearson’s *r*(337) = −0.36, *p* = 9.5e−12; visual cortex: Pearson’s *r*(337) = −0.26, *p* = 9.0e−7). SCZ = schizophrenia; PRS = polygenic risk score; RSFA = resting state functional amplitude. *q ≤ 0.05. Error bars = standard error.

Computational rodent work suggests the relative presence of SST and PVALB interneurons is a determinant of functional differences and hierarchical organization across cortex^11^. Spiking activity in cortex progresses from shorter timescales in sensory and unimodal cortices to longer timescales in association and integrative cortices^15,37^. This functional organization may be indexed by variability in the resting-state BOLD signal. Accordingly, we examined whether the difference of cortical *SST* and *PVALB* expression covaries with an in-vivo measurement of cortical signal variability, resting-state functional amplitude (RSFA). Voxel-wise RSFA was calculated using the UK Biobank sample (*n* = 9713) and averaged across the 400 parcel functional atlas of Schaefer and colleagues (Fig. 4a)^32^. Between-subject hierarchical clustering was conducted to reduce data dimensionality and identify cortical territories with similar patterns of signal amplitude across individuals (Fig. 4b). A seven-cluster solution was selected, corresponding to limbic A (light beige), limbic B (dark beige), cingulo-opercular (teal), temporo-parietal (orange), prefrontal (red), somato/motor (blue), and visual (purple) clusters. With the exception of Figs. 5a, b and 6b, clusters serve to aid in visualization and do not influence statistics. Consistent with recent work^38^, this data-driven dimensionality reduction broadly sorted association and unimodal aspects of cortex.

Computational models suggest that increased PVALB density may correspond to greater recurrent inhibition of excitatory neurons and faster timescales (i.e., higher RSFA). Likewise, increased relative presence of *SST* is predicted to correspond to longer timescales of activity (i.e., lower RSFA)^11^. Earlier work documents a correlation of interneuron marker expression with fractional Amplitude of Low-Frequency Fluctuations (fALFF)^8^, a metric closely tied to RSFA, within a circumscribed set of cortical areas. Consistent with these predictions, a negative spatial correlation was observed between RSFA and the relative difference of *SST* and *PVALB* expression (Fig. 4c; *r*(337) = −0.53, *p* < 2.2e−16; *r*_s_ = −0.60, *p* < 2.2e−16). Across 152,207,628 two-gene pairings, the *SST*–*PVALB* to RSFA correlation (*r* = −0.53; Fig. 4c) was the 54,303rd most negatively associated pair (top 0.04%). Analysis of deconvolved cell fractions across cortex revealed that SST had the strongest negative spatial association to RSFA (Fig. 4d; frontal cortex signature: *r*(337) = −0.48, *p* < 2.2e−16; visual cortex signature: *r*(337) = −0.36, *p* = 1.2e-11), while PVALB cell fractions were the most positively correlated (frontal cortex signature: r(337) = 0.47, p < 2.2e−16; visual cortex signature: *r*(337) = 0.34, *p* = 1.7e−10), relative to all other cell types. Relative difference of SST and PVALB cell fractions was the most associated with RSFA, relative to all pairwise cell combinations from frontal (*n* = 272) and visual (*n* = 240) cortex (Fig. 4e). Overall, these analyses use *ex-vivo* transcriptional data to identify SST and PVALB interneurons as cellular correlates of in-vivo measures of cortical signal variability (i.e., RSFA).

### Polygenic variation among *PVALB*-correlated genes underlies cortical brain function

Genome-wide association studies (GWAS) demonstrate that the genetic bases of many complex traits are due to the cumulative weight of genetic variants spread across the entire genome, each with a subtle effect^39^. Brain phenotypes such as resting-state functional amplitude likely possess a similar polygenic architecture. However, functionally-relevant polymorphisms can cluster in genes expressed within associated tissue and cell types^40^. We tested whether single-nucleotide polymorphisms (SNPs) that explain heritable variance in brain activity (i.e., RSFA) are enriched within genes linked to *PVALB* and *SST*, which would provide insight into the molecular basis of the resting-state BOLD fluctuations.

We established the SNP heritability of RSFA. A significant proportion of between-subject variation in cluster-wise RSFA was due to common genetic variants [Fig. 5a; $h_{{\mathrm{snp}}}^2$: limbic A = 0.25 (SE 0.04), limbic B = 0.30 (SE 0.04), cingulo-opercular = 0.21 (SE 0.04), temporo-parietal = 0.29 (SE 0.04), prefrontal = 0.33 (SE 0.04), somato/motor = 0.21 (SE 0.04), visual = 0.07 (SE 0.04)]^41^. See Supplementary Fig. 11 for parcel-wise heritability estimates. Interneuron-correlated gene sets were nominated using a “guilt-by-association” logic. That is, genes that were spatially correlated to interneuron markers (i.e., *SST*, *PVALB*) were assumed to relate to each interneuron subtype. Using cortical AHBA data, genes were rank-ordered by their spatial correlation to each interneuron marker and the top 500 most-correlated genes were selected. Interneuron-related SNP lists were generated for each gene set by identifying variants within ±5000 base pairs from transcription start and stop site of each gene. *PVALB* and *SST* SNP sets were non-overlapping. The eQTL variants for each gene set were included, defined using cortical data from the CommonMind consortium^42^ and NIH GTEx^43^. We denote the SNP lists for each interneuron gene set as PVALB_SNP_ and SST_SNP_ (see Supplementary Data). Genetic relatedness matrices were calculated for the UKB sample using each SNP set, and heritability was estimated using GCTA-GREML simultaneously across three partitions: PVALB_SNP_, SST_SNP_, and a partition containing all remaining genotyped variants^41^.

Indicating that genetic variance in RSFA, a measure of in-vivo functional variability, is explained by genes linked to *PVALB* interneurons, the PVALB_SNP_ set accounted for a significant proportion of heritable variance of the temporo-parietal [$h_{{\mathrm{PVALB}}}^2 = 0.036$ (SE 0.010), *q* = 0.0007], prefrontal [$h_{{\mathrm{PVALB}}}^2 = 0.022$ (SE 0.0091), *q* = 0.015], and somato/motor [$h_{{\mathrm{PVALB}}}^2 = 0.021$ (SE 0.0090), *q* = 0.015], visual [$h_{{\mathrm{PVALB}}}^2 = 0.020$ (SE 0.0089), *q* = 0.021], limbic A [$h_{{\mathrm{PVALB}}}^2 = 0.016$ (SE 0.009), *q* = 0.04], limbic B [$h_{{\mathrm{PVALB}}}^2 = 0.026$ (SE 0.009), *q* = 0.007] RSFA clusters, but at only trend-level in cingulo-opercular cluster [$h_{{\mathrm{PVALB}}}^2 = 0.014$ (SE = 0.0090), *q* = 0.053]. Conversely, the SST_SNP_ set did not explain a significant proportion of heritable variance across any partition ($h_{{\mathrm{SST}}}^2$’s < 0.011, *q*’s > 0.44). We next tested whether the PVALB_SNP_ set explains more genetic variance in RSFA than would be expected by chance. Enrichment was calculated as the proportion of heritability explained by the SNP partition, divided by the fraction of SNPs in that partition (>1 denotes enrichment). We observed enrichment greater than 1 for visual (enrich = 8.10, SE = 0.31), somato/motor (enrich = 3.56, SE = 0.32), temporo-parietal (enrich = 4.10, SE = 0.34), prefrontal (enrich = 2.36, SE = 0.32), cingulo-opercular (enrich = 2.04, SE = 0.31), limbic A (enrich = 1.98, SE = 0.32), and limbic B (enrich = 3.41, SE = 0.33) clusters. The PVALB_SNP_ list (*N* = 9571 variants) constituted 2.8% of total analyzable genotyped SNPs (*N* = 337,501 variants), but accounted for 5.6–22.9% (*M* = 10.4, SD = 6.0) of total genetic variance across each of the RSFA clusters (Fig. 5a). The SST_SNP_ partition (2.5% of available variants) only explained an enriched proportion of variance within the cingulo-opercular (enrich = 1.86, SE = 0.33) RSFA cluster.

An important unanswered question is whether the genetic determinants of RSFA are uniform across cortex, or whether they vary according to underlying transcriptional patterns. We tested whether the PVALB_SNP_ and SST_SNP_ partitions explain a greater percentage of heritable RSFA variance in regions where the respective marker is most expressed. Partitioned heritability was calculated across 400 Schaefer cortical parcels. Across all parcels with AHBA expression data, normalized genetic variance explained by the PVALB_SNP_ partition was positively correlated to *PVALB* expression (Fig. 5c; *r*(326) = 0.35, *p* = 1.04e−10; *r*_s_ = 0.40, *p* = 2.2e−14), corresponding to visual, superior temporal, and parietal cortex. Across all genes, *PVALB* was the 115th most positively correlated gene (top 0.66% of 17,448 transcripts; Spearman = 29th/17,448 = 0.17%) to the PVALB_SNP_ partition map, indicating that this positive relationship is not obligated by global statistical properties (Fig. 5d). This relationship remained significant after controlling for the overall SNP heritability of each parcel (*β* = 0.22, *t*(336) = −4.70, *p* = 3.9e−6). Conversely, PVALB_SNP_ heritability was negatively correlated to *SST* expression (*r*(326) = −0.39, *p* = 6.32e−14; *r*_s_ = −0.37, *p* = 6.5e−12). There was not a significant parcel-wise relationship between SST_SNP_ partitioned heritability and *SST* gene expression (Fig. 5f; *r*(326) = −0.017, *p* = 0.75; *r*_s_ = −0.021, *p* = 0.71). Converging with single-gene analyses, deconvolved estimates of PVALB cell fractions were the most positively correlated to PVALB partitioned heritability (Fig. 5e; frontal cortex: *r*(329) = 0.35, *p* = 2.27e−11; *r*_s_ = 0.39, *p* = 2.2e−13; visual cortex: *r*(329) = 0.31, *p* = 6.67e−9; *r*_s_ = 0.34, *p* = 1.5e−10). These results were highly consistent across parallel techniques using linkage-disequilibrium score regression (LDSC) and estimates of heritability from GWAS analyses of RSFA (Supplementary Fig. 12)^44^. Cumulatively, these data indicate that the heritable basis of resting-state functional amplitude is spatially heterogeneous, demonstrating a particularly important role of genes co-expressed with *PVALB*.

### Functional associates of schizophrenia genetic risk correlate with *PVALB* expression

Convergent evidence from animal models and post-mortem tissue analyses suggests that interneuron dysfunction is a core pathophysiological feature of schizophrenia^21^. To determine whether interneuron-related genetic variation is tied to disease liability, we tested whether polygenic risk for schizophrenia^45^ is greater among PVALB_SNP_ and SST_SNP_ variants, relative to the rest of the genome. Using a partitioned MAGMA analysis^46^, we divided rank-ordered *PVALB* and *SST* gene lists into bins of 500. We observed significant enrichment of schizophrenia polygenic risk for the top *PVALB* gene set (*β* = 0.094, *p* = 0.022), but not the top *SST* set (*β* = −0.001, *p* = 0.51). Suggesting that polygenic schizophrenia risk is greater among interneuron-related genes, we examined 20 gene bins and found that the enrichment of schizophrenia genetic risk decreased as gene sets became less spatially correlated with *PVALB* (*r*_s_ = −0.48, *p* = 0.03), but not *SST* (*r*_s_ = 0.027, *p* = 0.91; Fig. 6a).

To test whether polygenic risk for schizophrenia relates to cortical RSFA, we calculated schizophrenia polygenic risk scores (SCZ-PRS)^47^ using genotyped variants from individuals in the UK Biobank imaging sample. Across the seven RSFA clusters, SCZ-PRS negatively predicted RSFA in the visual cluster (Benjamini–Hochberg corrected *q* = 0.04; Fig. 6b; GWAS threshold *p* < 1.0), as well at trend-levels in somato/motor (*q* = 0.08) and prefrontal (*q* = 0.10) clusters. Consistent with the hypothesized link between PVALB interneurons and psychotic illness, the relationship between RSFA and polygenic schizophrenia risk was negatively correlated to *PVALB* expression across cortex (Fig. 6c; *r*(337) = −0.33, *p* = 3.1e−10; *r*_s_ = −0.35, *p* = 5.5e−11). That is, regions with greater *PVALB* expression (e.g., motor and visual parcels) showed stronger negative relationships between SCZ-PRS and RSFA. This relationship remained significant after controlling for overall SNP heritability of each parcel (*β* = −0.28, *t*(336) = −5.32, *p* = 1.95e−7), indicating that the effect isn’t driven by parcel-wise explainable genetic variance. Comparing the RSFA-schizophrenia polygenic risk map to all genes, *PVALB* was the among the top 0.83% negatively correlated expression profiles (145 out of 17,448; Spearman = 105/17,448 = 0.60%), showing that this relationship is not statistically obligated. Across all deconvolved cell types, parcel-wise PVALB cell fractions were on average the most negatively spatially correlated to schizophrenia risk RSFA effects (Fig. 6d; frontal cortex: *r*(337) = −0.36, *p* = 9.5e−12; *r*_s_ = −0.35, *p* = 2.4e−11; visual cortex: *r*(337) = −0.26, *p* = 9.0e−7; *r*_s_ = −0.26, *p* = 1.3e−6). Ontological enrichment analysis further revealed that the top 500 genes correlated with *PVALB* in the AHBA data contained genes associated to schizophrenia, neuronal signaling, and gated channel activity (Table 1). Together, these data suggest that schizophrenia-related genetic variants are associated with cell types, particularly parvalbumin interneurons, and could explain functional disruptions across cortex.

**Table 1:** Enrichment terms for interneuron-correlated genes.

Gene list	Category	ID	Name	*p*	*q* FDR-BH	Hits	Genes in GO
SST	GO: BP	GO:0099536	Synaptic signaling	1.37e−5	3.04e−2	37	687
SST	GO: BP	GO:0099537	Trans-synaptic signaling	2.43e−5	3.04e−2	36	678
SST	GO: CC	GO:0097458	Neuron part	1.26e−6	6.83e−4	66	1545
SST	GO: CC	GO:0098793	Presynapse	4.03e−4	3.64e−2	20	341
PVALB	GO: MF	GO:0005261	Cation channel activity	3.33e−12	1.74e−9	33	306
PVALB	GO: MF	GO:0005249	Voltage-gated potassium channel activity	1.33e−10	1.69e−8	17	91
PVALB	GO: BP	GO:0071805	Potassium ion transmembrane transport	1.53e−9	2.43e−6	21	181
PVALB	GO: BP	GO:0098655	Cation transmembrane transport	3.23e−9	3.09e−6	48	738
PVALB	GO: CC	GO:0034703	Cation channel complex	1.04e−10	5.07e−8	23	176
PVALB	GO: CC	GO:0034702	Ion channel complex	3.03e−10	5.07e−8	29	291
PVALB	Disease	C0036341	Schizophrenia	5.85e−7	1.6e−3	68	1561

Ontological enrichment analyses were conducted with ToppGene on the 500 genes used to generate the PVALB_SNP_ and SST_SNP_ lists. See Supplementary Data for full enrichment tables.

## Discussion

Integrating genetic, transcriptional, and neuroimaging data, we demonstrate that spatial distributions of interneurons are stereotyped across species and development, and explain a substantial portion of the heritable variation in RSFA, a measure of in-vivo brain activity. Somatostatin and parvalbumin interneuron markers were negatively spatially correlated across cortex, a relationship that was robust in early developmental periods in humans and evolutionarily conserved in non-human primates (Fig. 1). Stereotyped patterns of *SST* and *PVALB* expression were observed in subcortex and were consistent with cell density estimates in rodents (Fig. 3). Computational models theorize that interneuron ratios underlie regional differences in cortical brain function^11^. Providing empirical support for this hypothesis, relative *SST* and *PVALB* expression in post-mortem brain tissue aligned with spatial RSFA in the general population (Fig. 4). Indicating the functional relevance of this spatial relationship, genetic polymorphisms linked to PVALB correlated genes accounted for an enriched proportion of heritable variance underlying cortical signal amplitude (Fig. 5). Critically, the amount of in-vivo variance explained by PVALB-linked SNPs positively tracked spatial expression of *PVALB* in independent post-mortem brain tissue, suggesting that common genetic polymorphisms influence brain function in a cell-preferential and regionally variable manner. Implicating genetic differences among interneurons in schizophrenia, schizophrenia-related polygenic risk was enriched among genes co-expressed with interneurons, and predicted reduced resting-state functional amplitude across cortex, following the spatial landscape of *PVALB* gene expression (Fig. 6).

Adaptive functioning depends on the integration of information across timescales. Higher-order cognition often requires information accumulation over time, whereas sensorimotor processing entails rapid adaption to changing external stimuli^15,18^. These computational demands are met, in part, through the hierarchical organization of anatomic and functional connections in cortex, as well as cytoarchitectural differences across brain regions^10,48^. Our data indicate that interneuron ratios, as indexed by *SST* and *PVALB* expression, are an important feature of regionally variable brain function. Due to unique functional and synaptic properties of somatostatin and parvalbumin interneurons, relative shifts in their density can alter the balance of inhibitory control^11^. SST interneurons generally synapse onto dendrites of pyramidal neurons to gate incoming cortical signals, whereas PVALB interneurons generally provide perisomatic inhibition that is well-suited for feedback inhibition and output regulation^2^. Increased dendritic (i.e., SST) over perisomatic (i.e., PVALB) inhibition could lead to more robust filtering of task-irrelevant information, allowing for greater recurrent excitation and integration of information over time^19^. Conversely, parvalbumin-dense sensorimotor regions may benefit from fast responses and lower recurrent excitation to adapt to rapidly changing inputs^15^.

Our analyses provide molecular genetic support for a relationship between parvalbumin interneurons and the hemodynamic signal. BOLD signals are preferentially coupled to gamma-band oscillations (30–80 Hz) relative to other frequency domains^20^. Importantly, fast-spiking parvalbumin interneurons have been experimentally linked to gamma oscillations^49^. Here, we provide initial evidence in humans for the influence of parvalbumin interneurons on fMRI signal variability. For instance, polygenic variation among parvalbumin correlated genes explained upwards of 23% of the heritable variance in RSFA in visual cortex.

Schizophrenia is one of the most heritable forms of psychiatric illnesses (*h*^2^ ~81%)^50^, with converging lines of evidence pointing toward GABAergic and parvalbumin interneuron abnormalities as cardinal features of the disorder^21,51^. Patients with schizophrenia exhibit reduced levels of GAD67, an enzymatic precursor of GABA^52^, and are characterized by PVALB interneurons with atypical perineuronal nets^53^. These abnormalities are thought to underlie disorder-related disruption of gamma-band oscillations and working memory^51^. Linking these observations, we demonstrate that polygenic schizophrenia risk is increased among genes that are spatially correlated to *PVALB* (Fig. 6a), expanding upon cell transcriptomic work implicating cortical interneurons as an illness marker^40^. We further show a negative association between individual polygenic schizophrenia risk and RSFA in a large population-based sample (Fig. 6b). The topography of these effects follows the spatial profile of *PVALB* expression across cortex (Fig. 6c), highlighting the potential role of parvalbumin interneurons in mediating brain-based intermediate phenotypes associated with psychotic illness. This report also compliments evidence that schizophrenia risk gene expression is spatially correlated to disorder-related changes in brain morphology^54,55^.

Disruption of excitatory/inhibitory balance may reflect a cross-diagnostic marker of psychiatric illness^56^. For instance, decreased expression of parvalbumin cell markers is evident in both schizophrenia and bipolar disorder^57^, while major depressive disorder is marked by preferential reductions in somatostatin interneurons^5^. Delineating the region-specific roles of cortical interneuron subtypes will provide insight into cross-diagnostic patterns of both behavior and brain function. With regard to depressed mood, modulation of cortical somatostatin interneurons causally influences anxiety- and depression-like behavioral phenotypes in rodents^5,58^. In line with this observation, we observed preferential expression of somatostatin within mPFC, NAcc, mediodorsal thalamus, and VTA (Supplementary Fig. 9), a distributed set of regions implicated in reward and affective information processing^35^. Somatostatin-biased regions (ACC, mPFC, and insula) also correspond to areas of cortical thinning in patients with MDD and individuals reporting elevated negative affect^59–61^.

The present findings should be interpreted in light of several limitations. First, we use single molecular markers to infer the relative presence of SST and PVALB interneurons, which are not sensitive to morphological and physiological differences among interneuron subgroups^2^, a point we sought to address through the analyses of deconvolved cell type distributions. Second, we employ a “guilt-by-association” logic to nominate interneuron related gene sets. While we cannot conclude that genes within each identified interneuron group directly influence interneuron function, similar correlation-based nomination approaches have been shown to correspond well with a priori defined gene groups^62^. However, the examination of enrichment terms (Table 1 and Supplementary Information) allows for a more precise understanding of the biological processes contributing to these results. Third, our measure of brain signal amplitude (RSFA) is likely explained by a mixture of neural and vascular signals. However, effects related to BOLD variability have been shown to be stable even after controlling for measures of vasculature^63^, and vascular confounds are a caveat of many BOLD measures of brain function. Last, our in-vivo imaging and genetic analyses focus on an aging population of white British individuals. Future work should examine the stability of these results across diverse populations^64,65^.

Inherited genetic variation shapes brain function within and across individuals^66^. Analyses of spatially-dense, whole-genome, expression atlases increasingly reveal transcriptional correlates of brain function^8^, structure^67^, functional connectivity^6,7,9^, and psychiatric illness^54^. With the parallel emergence of large-scale imaging genetic data^23^, it is now possible to bridge structural genetic, transcriptional, and large-scale neuroimaging brain phenotypes. Here, we leverage these data to show that interneuron marker distributions are consistent across species, correlate with cortical signal amplitude, explain regional differences in heritable brain function, and associate with genetic risk for schizophrenia in the general population.

## Methods

### Allen Human Brain Atlas

Publicly available human gene expression data from six postmortem donors (1 female), aged 24–57 years (42.5 ± 13.4) were obtained from the Allen Institute^27^. Data reflect the microarray normalization pipeline implemented in March 2013 (http://human.brain-map.org) and analyses were conducted according to the guidelines of the Yale University Human Subjects Committee. Microarray probes from eight overarching ontological categories were selected: cortex, dorsal thalamus, striatum, globus pallidus, hypothalamus, hippocampus, amygdala, and the combined substantia nigra and ventral tegmentum (see Supplementary Information). Probes without Entrez IDs were removed. Probe-wise noise for each donor was quantified as the number of above-threshold samples in cortex, divided by total cortical sample count. A probe-wise average was computed across all six donors, which was used to remove probes expressed in fewer than 20% of cortical samples^68^. If more than one probe existed for a given gene, the one with the highest mean expression level was selected for further analysis, resulting in 17,448 brain-expressed genes.

Individual cortical tissue samples were mapped to each AHBA donor’s Freesurfer derived cortical surfaces, downloaded from Romero-Garcia and colleagues^69^. Native space midthickness surfaces were transformed to a common fsLR32k group space while maintaining the native cortical geometry of each individual donor. The native voxel coordinate of each tissue sample was mapped to the closest surface vertex using tools from the HCP workbench^70^. A cortical tissue sample was not analyzed if it was greater than 4 mm from the nearest surface vertex, resulting in 1683 analyzable cortical samples. Microarray expression of each gene was mean- and variance-normalized separately for each of the eight analyzed regions, revealing relative expression differences within cortical and subcortical territories. For region-wise expression analyses (e.g., Fig. 1c), ontological categories from the AHBA were used to calculate the median, min–max, and interquartile range of relative expression in each region. Detailed information about the analyzed regions is provided in the Supplementary Information. Cortical data visualization was carried out using *wb_view* from the HCP workbench^70^. The MNI locations of striatal and thalamic samples were cross-referenced to functional atlases of Choi and colleagues^71^ and Hwang and colleagues^72^. With AFNI, a single voxel (1 mm^3^) region of interest (ROI) was generated at the MNI location of each sample. A functional network label was assigned if the ROI fell within a volumetric parcel. If the sample did not overlap with the functional atlas, the associated ROI was expanded to 2 mm^3^ and the network with the most overlapping voxels in the ROI was assigned. If the expanded 2 mm^3^ ROI did not overlap, the process was repeated using a 3 mm^3^ ROI. A sample was omitted from analysis if the 3 mm^3^ ROI did not overlap with the associated functional atlas. Functional sub-regions with 3 or fewer samples were excluded from analyses. This process was repeated for coordinates aligned to MNI152 1 mm space using ANTs registration tools (https://github.com/chrisgorgo/alleninf/tree/master/alleninf/data).

Subcortical AHBA expression data was compared to rodent cell density counts published by Kim et al.^11^. *Z*-transformed AHBA expression values were summarized across major subdivisions of the seven subcortical regions analyzed (e.g., CA1, NAcc, etc.; Supplementary Data). Median AHBA expression values were used for analyses in Fig. 3b. Rodent homologs of each sub-region were manually identified and PVALB and SST cell densities were averaged across male and female samples.

### UKB imaging processing

Minimally preprocessed resting-state fMRI data from the UK Biobank were analyzed, reflecting the following preprocessing steps: motion correction with MCFLIRT^73^, grand-mean intensity normalization, highpass temporal filtering, fieldmap unwarping, and gradient distortion correction. Noise terms were identified and removed using FSL ICA+FIX. Full information on the UKB preprocessing is published^23^. Additional processing was conducted in AFNI^74^ and consisted of 3dDespike, resampling to MNI152 space using the UKB generated linear and nonlinear transforms, FWHM blur of 4.0 mm, regression of CSF, WM, and global resting state signals, and first- and second-order trend removal. Voxel-wise RSFA maps were generated with 3dRSFC and then averaged within each of the approximately symmetrical 400 volumetric parcels from the 7-network parcellation of Schaefer and colleagues^32^. Due to signal blurring between lateral striatum and insular cortex, supplemental resting-state functional connectivity analyses reflect an additional local white matter regression against gray matter using AFNI anaticor. Imaging analyses were conducted in volume, but visualized on the cortical surface. Resting-state functional connectivity between striatum, thalamus, and cortex was estimated using AFNI’s 3dNetCorr, which calculated the Fisher-*Z* transformed correlation values of timeseries across the Choi 7-region striatal atlas^71^, the Hwang 9-region thalamic atlas^72^, and the Schaefer 400-region cortical atlas^32^.

A total of 13,236 UKB subjects were processed through the imaging pipeline. Subjects with mean run-wise frame-to-frame head motion greater than 0.20 mm, and inverted resting-state SNR greater than three standard deviations above the mean were removed. After filtering for white British subjects with usable genetic data, cryptic relatedness <0.025, and conducting row-wise deletion for the variables age, sex, height, weight, BMI, three head position coordinates (*X*, *Y*, *Z*), combined gray/white matter volume, combined ventricular/CSF volume, diastolic and systolic blood pressure, run-wise resting state motion, resting state inverse SNR, T1 inverse SNR, and UK Biobank assessment center, 9713 subjects remained for analyses (percent female = 54.33, mean age = 63.67, SD = 7.45, min/max age = 45–80). We also included the anthropometric measures of height, BMI, weight, and blood pressure. Analyses were conducted according to the guidelines of the Yale University IRB.

### UKB genetics

UK Biobank genotype data was filtered to include only white British subjects with imaging data passing the quality control thresholds described above. Plink v1.9 was used to remove samples with missingness >0.10, SNPs with minor-allele frequency <0.05, Hardy–Weinberg equilibrium *p* < 1 × 10^−6^, and call rate <0.02, resulting in 337,501 autosomal variants^75^. GCTA software was used to calculate a genetic relatedness matrix to remove individuals with cryptic relatedness more than 0.025, leaving *N* = 9713 subjects for analysis^41^. Ten genetic principal components were then calculated for use as covariates in polygenic risk score and heritability analyses. When calculating polygenic risk for schizophrenia, SNPs from the major histocompatibility complex were censored except for the most significantly associated variant from the region.

### RSFA between-subject clustering and heritability

Voxel-wise RSFA data from the (*N* = 9713) UK Biobank sample was averaged within each of 400 roughly symmetric volumetric ROIs from the 7-Network cortical parcellation of Schaefer and colleagues^32^. Parcel-wise RSFA values were residualized for the effect of age, sex, age^2^, age × sex, age^2^ × sex, height, weight, BMI, combined gray/white matter volume (normed for head size), combined ventricular/CSF volume (normed for head size), diastolic and systolic blood pressure, run-wise rsfMRI motion, rsfMRI inverse SNR, T1 inverse SNR, three head position coordinates (*X*, *Y*, *Z*), and UK Biobank assessment center. Hierarchical clustering of residualized RSFA estimates was conducted using R in order to group regions with similar between-subject patterns of covariation. A 7-parcel RSFA clustering was selected. Raw RSFA values were then averaged across parcels falling within the same data-derived between-subject cluster for use in heritability analyses. SNP heritability of RSFA was estimated with genotyped data using GCTA-REML software. Age, sex, age^2^, height, weight, BMI, combined normed gray/white matter volume, combined normed ventricular/CSF volume, diastolic and systolic blood pressure, run-wise rsfMRI motion, rsfMRI inverse SNR, T1 inverse SNR, head coordinates (*X*, *Y*, *Z*), UK Biobank assessment center, and 10 genetic principal components were included as covariates.

Partitioned heritability analyses were conducted for the seven RSFA clusters and for each of the 400 individual cortical parcels. Using AHBA expression data, genes were rank ordered by their spatial cortical correlation to *SST* and *PVALB*. Genes without Entrez IDs were removed. The BioMart package^76^ was used to identify each gene’s transcription start and stop sites (±5000 base pairs) according to the GRCh37-hg19 genome assembly. Otherwise, the gene was cross-referenced to cortical eQTL databases from the NIH GTEx project^43^ and CommonMind consortium^42^. Intragenic (±5000 base pairs) and eQTL SNPs associated with the top 500 *SST* (*N*_SNP_ = 8308) and *PVALB* (*N*_SNP_ = 9571) correlated genes were used for partitioned heritability analyses, respectively denoted SST_SNP_ and PVALB_SNP_. A small subset of genes in each bin did not have analyzable variants using these criteria and thus did not contribute to results. Genetic-relatedness matrices for the *SST*_SNP_ and *PVALB*_SNP_ partitions were generated, as well as one for all remaining genotyped SNPs. RSFA heritability accounted for by each genetic relatedness matrix was estimated simultaneously for each of the three partitions using GCTA^41^. Partitioned heritability was then defined as the phenotypic variance explained by either SST_SNP_ or PVALB_SNP_, divided by the total phenotypic variance. To calculate the significance of individual partitions, we consider the Wald test statistic against the null of $h_{{\mathrm{part}}}^2 = 0$, which follows a half-half mixture of $\chi _0^2$ (a *χ*^*2*^ distribution with a probability mass at zero) and $\chi _1^2$ (a *χ*^2^ distribution with 1 degree of freedom). Enrichment values were calculated to determine if the proportion of variability explained by a partition was greater than the proportion of variants within the partition, defined as:

1 $${\mathrm{enrich}}_{{\mathrm{part}}} = \frac{{(h_{{\mathrm{part}}}^2/h_{{\mathrm{total}}}^2)}}{{(g_{{\mathrm{part}}}/g_{{\mathrm{total}}})}}$$

where $h_{{\mathrm{part}}}^2$ is the heritable variance explained by the SNP partition (e.g., PVALB_SNP_), $h_{{\mathrm{total}}}^2$ is the heritable variance explained by all partitions, *g*_part_ is the number of variants within the SNP partition, and *g*_total_ is the total number of genotyped SNPs. Standard error for SNP partitions were similarly scaled by the genome partition denominator. When calculating RSFA partitioned heritability across individual parcels (i.e., Fig. 5), those with outlier partitioned heritability (i.e., PVALB_PART_, SST_PART_) and expression (i.e., *PVALB, SST*) greater than 4 standard deviations from the mean were excluded, resulting in 328 observations across cortex. The spatial relationship between partitioned heritability estimates and *ex-vivo* AHBA gene expression patterns was then quantified using correlation (Fig. 5).

GCTA heritability results were replicated using LDSC regression^44^. Preprocessing of UKB imputed genetic data included censoring of SNPs with imputation accuracy estimate less than 90% (i.e., INFO < 0.9), minor allele frequency <0.01, Hardy–Weinberg equilibrium *p* < 1 × 10^−6^, call rate <0.1, genotype rate <0.1, and removal of non-biallelic variants using Plink v2.0. Genome-wide association analyses were conducted using the linear regression form of GCTA’s fastGWA utility^77^. The same quantitative and categorical covariates were used across GCTA and GWAS analyses. LDSC based estimates of RSFA heritability were conducted on 1,158,800 HapMap3 variants that overlapped with variants in the UKB GWAS summary statistics, using precomputed LD scores from 1000 Genomes European data (i.e., “*eur_w_ld_chr”*). Partitioned RSFA heritability analyses were conducted by examining SNPs occurring near the coding regions of the 500 genes most spatially correlated to *PVALB* and *SST* (i.e., two non-overlapping 500-gene bins). SNP locations were mapped to genes using the *biomaRt* package in R and AHBA Entrez IDs (i.e., GRCh37 build)^76^. SNP annotation files encompassed genetic variants occurring ±10,000 base pairs from the start–stop positions of each gene. A small subset of genes in each bin did not have analyzable variants using these criteria and thus did not contribute to results. The LDSC “*make_annot.py*” tool was used to create annotation files, filtering on HapMap3 variants and using 1000 Genomes Phase 1 genetic data to estimate LD. Partitioned heritability of the SST and PVALB SNP sets was estimated in conjunction with a full baseline model of 53 annotations (i.e., “*1000G_Phase1_baseline_ldscores”*), using precomputed allele frequencies (i.e., *1000G_frq*) and weights (i.e., “*weights_hm3_no_hla”*). These analyses provide a measure of genetic variance explained by the SST and PVALB SNP bins, conditioned on the baseline annotation model to prevent non-specific genetic signals from inflating stratified heritability estimates.

To assess whether schizophrenia polygenic risk was enriched among *SST* and *PVALB* correlated gene sets, competitive gene-set analysis was conducted using MAGMA^46^. Rank-ordered *SST* and *PVALB* genes were divided into twenty non-overlapping 500-gene bins. Schizophrenia summary statistics from the GWAS of Ripke and colleagues^45^ were used. Intragenic variants were defined using a ±5000 base pair window, and gene set enrichment was estimated simultaneously across all 40 gene bins, revealing whether a particular bin is more associated with polygenic risk for schizophrenia than all other genes. Polygenic risk for schizophrenia^45^ was calculated using PRSice^47^. Only the top-SNP from the major histocompatibility complex was used for the generation of individual risk scores. Benjamini–Hochberg false-discovery rate correction was conducted separately for each GWAS *p*-value threshold examined (e.g., correction for seven tests at the GWAS *p* < 1.0 threshold).

### NIH Blueprint processing

Publicly available microarray data from six adult macaque primates (three female) were downloaded from the Gene Expression Omnibus website (https://www.ncbi.nlm.nih.gov/geo; accession number GSE31613)^78^. Expression values were converted from log10 to log2. Data from two macaques (one female) were excluded due to sparse sampling across cortex. Samples from the following 10 cortical regions were included in our analyses: OFC, ACC, medial temporal lobe, temporal area, DLPFC, A1C, S1C, M1C, V1, and V2. The *collapseRows* function was used in R to select the probe with the highest mean expression and ComBat was used to remove residual donor effects. *SST* and *PVALB* expression were mean and variance-normalized to reveal relative expression differences across cortex.

### BrainSpan processing

Publicly available RNAseq reads per kilobase per million (RPKM) data from the Brainspan atlas were used to characterize patterns of interneuron-marker gene expression across development. Cortical tissue samples were analyzed from early fetal [8–12 post-conception weeks (pcw), donors = 10, samples = 88], early/mid fetal (13–21 pcw, donors = 10, samples = 88), late fetal (24–37 pcw; donors = 5, samples = 27), early infancy (4 months; donors = 3, samples = 22), late infancy (10 months; donors = 1, samples = 8), early childhood (1–4 years; donors = 5, samples = 41), mid/late childhood (8–11 years; donors = 2, samples = 30), adolescence (13–15 years; donors = 2, samples = 14), and adulthood (18–40 years; donors = 8, samples = 85) developmental stages. RNAseq probes without Entrez IDs were excluded and duplicated probes were removed by selecting the probe with the highest mean expression. Data was log2 transformed and the effect of donor was removed separately for each age group using ComBat^79^. Gene expression was then mean- and variance-normalized across cortical tissue samples separately for each developmental stage. When multiple ages were present in a development stage, age was included as a covariate in a linear regression predicting normalized *SST* expression from normalized *PVALB* expression.

### Single-cell analysis and deconvolution

Single-nucleus droplet-based sequencing (snDrop-seq) data from Lake and colleagues^31^ was obtained from the Gene Expression Omnibus website (“GSE97930” [https://www.ncbi.nlm.nih.gov/geo]). Count matrices derived from unique molecular identifier (UMI) were analyzed, reflecting 19,368 cells from visual cortex (BA17) and 10,319 cells from frontal cortex (BA10 and BA6) across six postmortem adult brains. Collinearity among transcriptionally similar cell types was reduced by labeling cells according to 18 superordinate cell identities defined by Lake and colleagues^31^. Single-cell data were preprocessed using Seurat^80^. After checking for outlier cells and minimally expressed genes, default global-scaling normalization was applied (i.e., “LogNormalize”). Entrez IDs and gene symbols were used to cross-reference single-cell and AHBA data, and genes lacking matches or with more than one match were removed. Non-log transformed data were then analyzed using CIBERSORTx^22^ to impute cell type fractions present in the AHBA data. Independent gene signature matrices were defined with visual cortex and frontal cortex cell data. Cell type fractions were then estimated separately for each AHBA donor, once using a visual cell signature and once using a frontal cortex cell signature. Cell type fractions for each AHBA cortical sample were mapped to the cortical surface and summarized among Schaefer^32^ atlas ROIs in the same manner as single-gene expression data.

### Replication analyses

In this paper, we sought to replicate findings with alternative methodologies and independent datasets whenever possible. In Fig. 1, we document a negative spatial relationship between SST and PVALB interneuron markers in cortex, using microarray data from the AHBA. This effect was replicated using RNASeq data from the AHBA (Supplementary Fig. 4), in an independent sample from the Brainspan Atlas (Fig. 1g), in cortex of non-human macaque primates (Fig. 1d, f), and with a complementary polygenic technique implemented with single-cell data and CibersortX (Fig. 2). We also identify stereotyped patterns of *SST* and *PVALB* expression in subcortical territories, which were replicated with independent measures of cell density in rodents (Fig. 3). Spatial associations between cortical function (i.e., RSFA) and markers of interneurons were also established with two techniques. The first approach examined highly cell-specific individual gene markers and the second approach utilized polygenic cellular deconvolution based on multivariate transcriptional signatures of cell types (Fig. 4). Further, heritability and partitioned heritability analyses of cortical RSFA were replicated with two techniques, GCTA and LD score regression (Fig. 5 and Supplementary Fig. 12). These methods yielded highly convergent results that were robust to genetic preprocessing choices (e.g., analysis of genotyped versus imputed variants) and model assumptions of underlying genetic architecture.

### Reporting summary

Further information on research design is available in the Nature Research Reporting Summary linked to this article.

## Supplementary information

Supplementary Information

Description of Additional Supplementary Files

Supplementary Data 1

Reporting Summary

## Data Availability

The data that support these findings are either publicly available, provided with the paper, or are under third party restrictions. Publicly available data are accessible at the following locations: Allen Human Brain Atlas (https://human.brain-map.org/), UK Biobank (https://www.ukbiobank.ac.uk/), Brainspan Atlas of the Developing Human Brain (https://www.brainspan.org/), NIH Blueprint NHP Atlas (https://www.blueprintnhpatlas.org/), NIH GTEx (https://commonfund.nih.gov/gtex), CommonMind (https://www.nimhgenetics.org/resources/commonmind), snDrop-seq data at Gene Expression Omnibus “GSE97942” (https://www.ncbi.nlm.nih.gov/geo/), and GWAS data from the Psychiatric Genomics Consortium (https://www.med.unc.edu/pgc/). Data not under third-party restrictions are available at https://github.com/HolmesLab/2020_NatComm_interneurons_cortical_function_schizophrenia.

## Code availability

Code not under third-party restrictions are available at https://github.com/HolmesLab/2020_NatComm_interneurons_cortical_function_schizophrenia.
