# Supplementary material for: Transcriptional and imaging-genetic association of cortical interneurons, brain function, and schizophrenia risk
Source: Nat Commun. 2020 Jun 8;11:2889. doi: 10.1038/s41467-020-16710-x (PMC7280213; doi:10.1038/s41467-020-16710-x)
Supplement: Supplementary file 1 — Supplementary Information [file 41467_2020_16710_MOESM1_ESM.pdf]

SUPPLEMENTARY INFORMATION

Supplementary Data: Transcriptional and imaging-genetic association of cortical interneurons, brain function, and schizophrenia risk

Authors: Kevin M Anderson[1], Meghan A Collins[1], Rowena Chin[1], Tian Ge[2,3], Monica D Rosenberg[1,4], Avram J Holmes[1,3,5*]

[1]Department of Psychology, Yale University, New Haven, Connecticut 06520
[2]Psychiatric and Neurodevelopmental Genetics Unit, Center for Genomic Medicine, Massachusetts General Hospital, Boston, MA 02114, USA
[3]Department of Psychiatry, Massachusetts General Hospital, Harvard Medical School, Boston, MA 02114, USA
[4]Department of Psychology, University of Chicago, Chicago, IL 60637
[5]Department of Psychiatry, Yale University, New Haven, Connecticut 06520, USA
*Corresponding author.

Correspondence: Avram J. Holmes, Yale University, Department of Psychology, 402 Sheffield Sterling Strathcona Hall, 1 Prospect Street, New Haven, CT 06511, Phone: 203-436-9240, Email: avram.holmes@yale.edu.

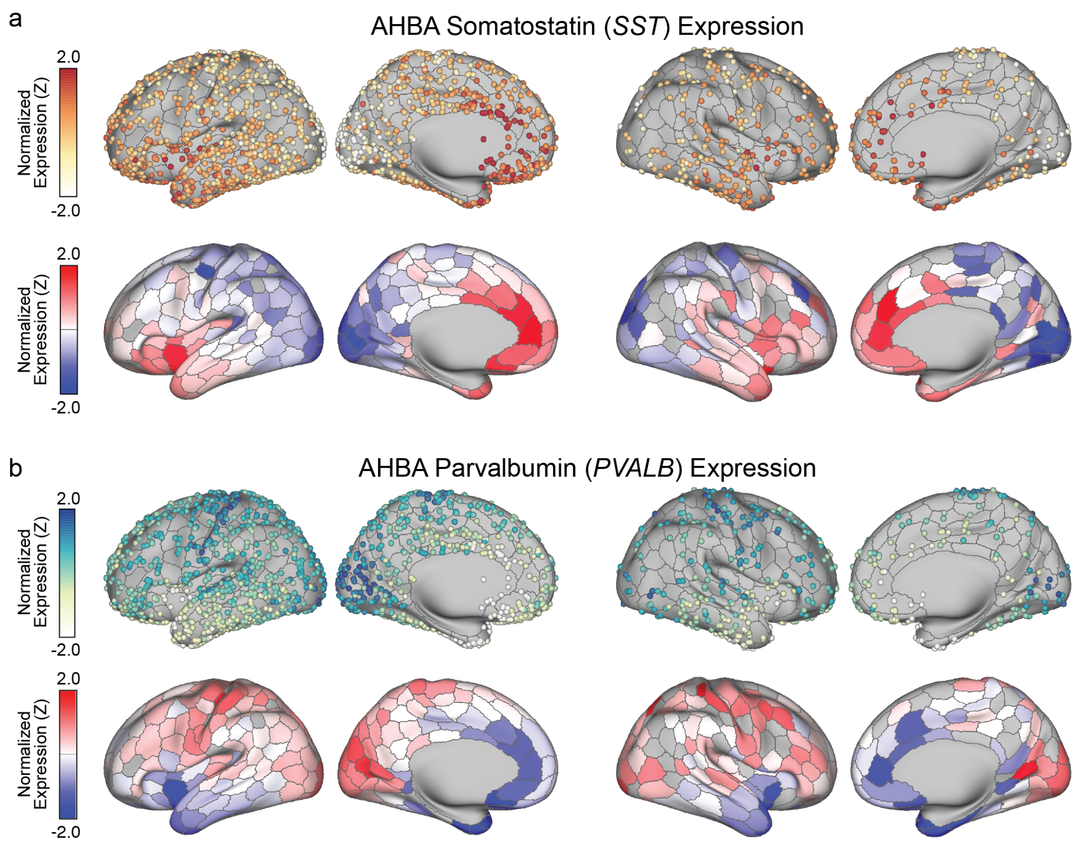

Supplemental Figure 1. Sample and parcel-wise expression of interneuron markers (a) *SST* and (b) *PVALB*. Gene-expression was z-transformed separately for each AHBA donor, projected onto individualized cortical surfaces from Freesurfer, and aligned to a group atlas template. Each dot represents an individual AHBA samples (top panels), which were averaged within each of 400 roughly symmetric parcels from Schaefer and colleagues[1]. Gray parcels in the bottom panel reflect lack of overlapping AHBA samples.

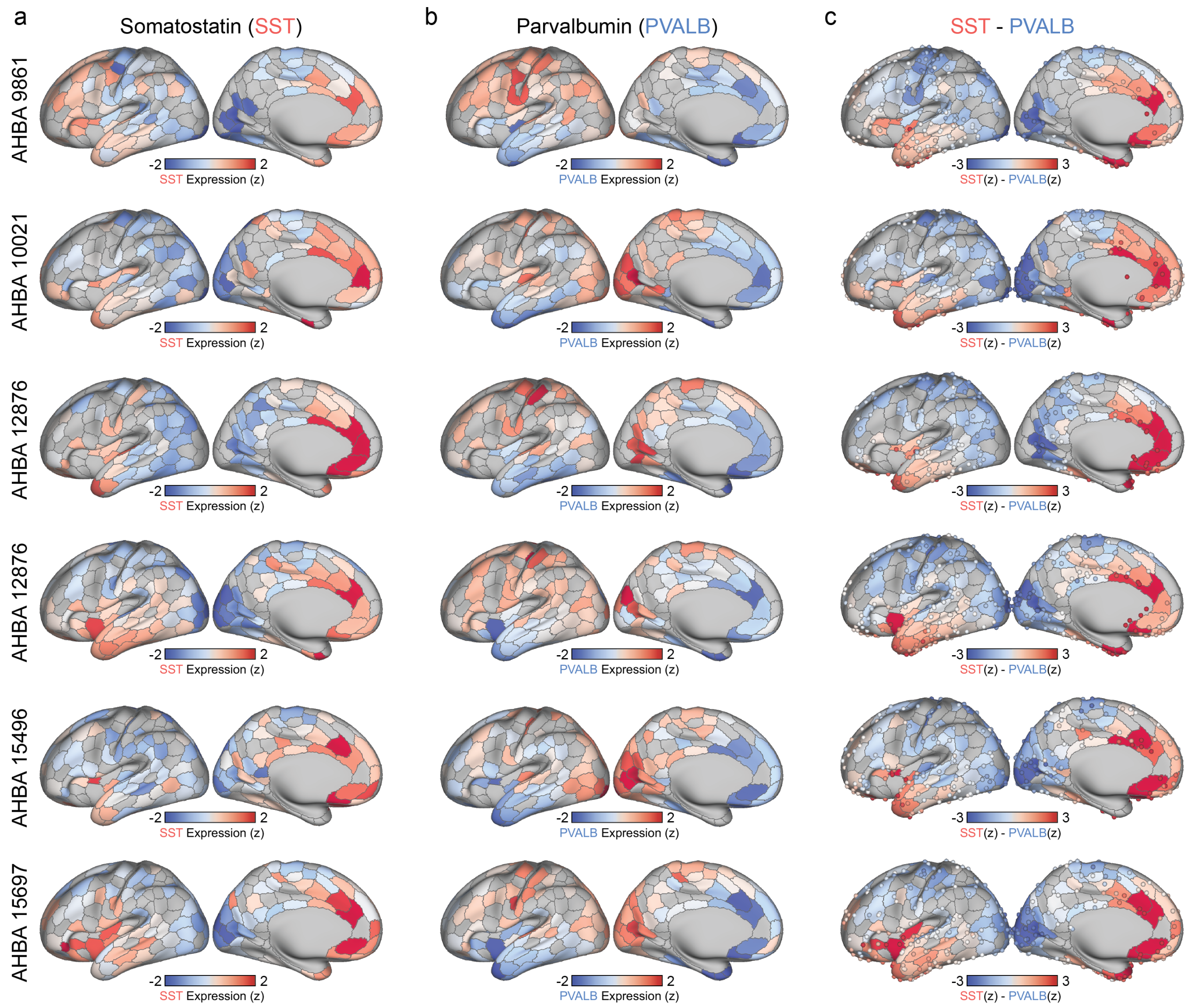

Supplemental Figure 2. Consistent *SST* and *PVALB* expression gradients across individuals. Average (a) *SST* and (b) *PVALB* expression within each left hemisphere parcel, presented individually across the six AHBA donors. For each donor, gene expression across cortical samples were mean- and variance-normalized before being averaged in each individual parcel. Gray areas denote the lack of available data. (c) The difference between normalized *SST* and *PVALB*. Red denotes greater relative presence of *SST* and blue denoted greater relative presence of *PVALB*. Individual tissue samples are also plotted on the cortical surface and colored by relative *SST*-*PVALB* expression to illustrate sample coverage for each region.

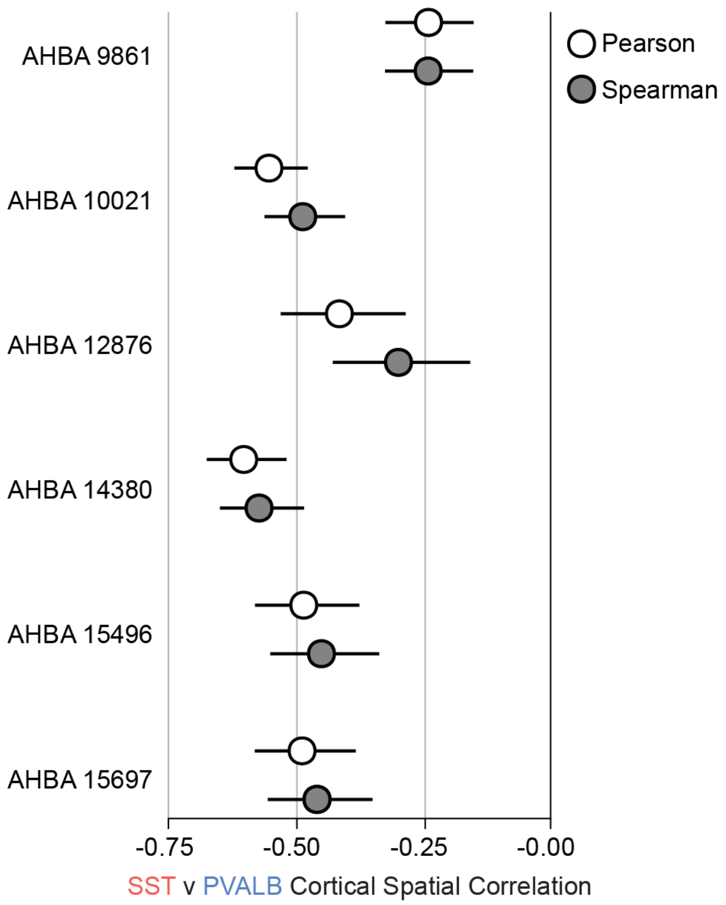

Supplementary Figure 3. Sample-wise correlation between *SST* and *PVALB* for each individual AHBA donor. White and gray dots denote Pearson and Spearman correlations, respectively. Error bars denote 95% confidence intervals. A significant negative correlation between *SST* and *PVALB* was observed for all donors: AHBA 9861 [r(453)=-0.24, p=2.0e-7; r_s=-0.24, p=1.90e-7], AHBA 10021 [r(355)=-0.55, p<2.2e-16; r_s=-0.49, p<2.2e-16] AHBA 12876 [r(173)=-42, p=1.0e-8; r_s=-30, p=5.6e-5], AHBA 14380 [r(253)=-0.60, p<2.2e-16; r_s=-0.57, p<2.2e-16], AHBA 15496 [r(212)=-0.49, p=4.2e-14; r_s=-0.45, p=3.8e-12], and AHBA 15697 [r(225)=-49, p=4.3e-15; r_s=-0.46, p=2.7e-13]. Number of independent cortical samples for each donor: AHBA 9861=455, AHBA 10021=357, AHBA 12876=175, AHBA 14380=255, AHBA 15496=214, AHBA 15697=227.

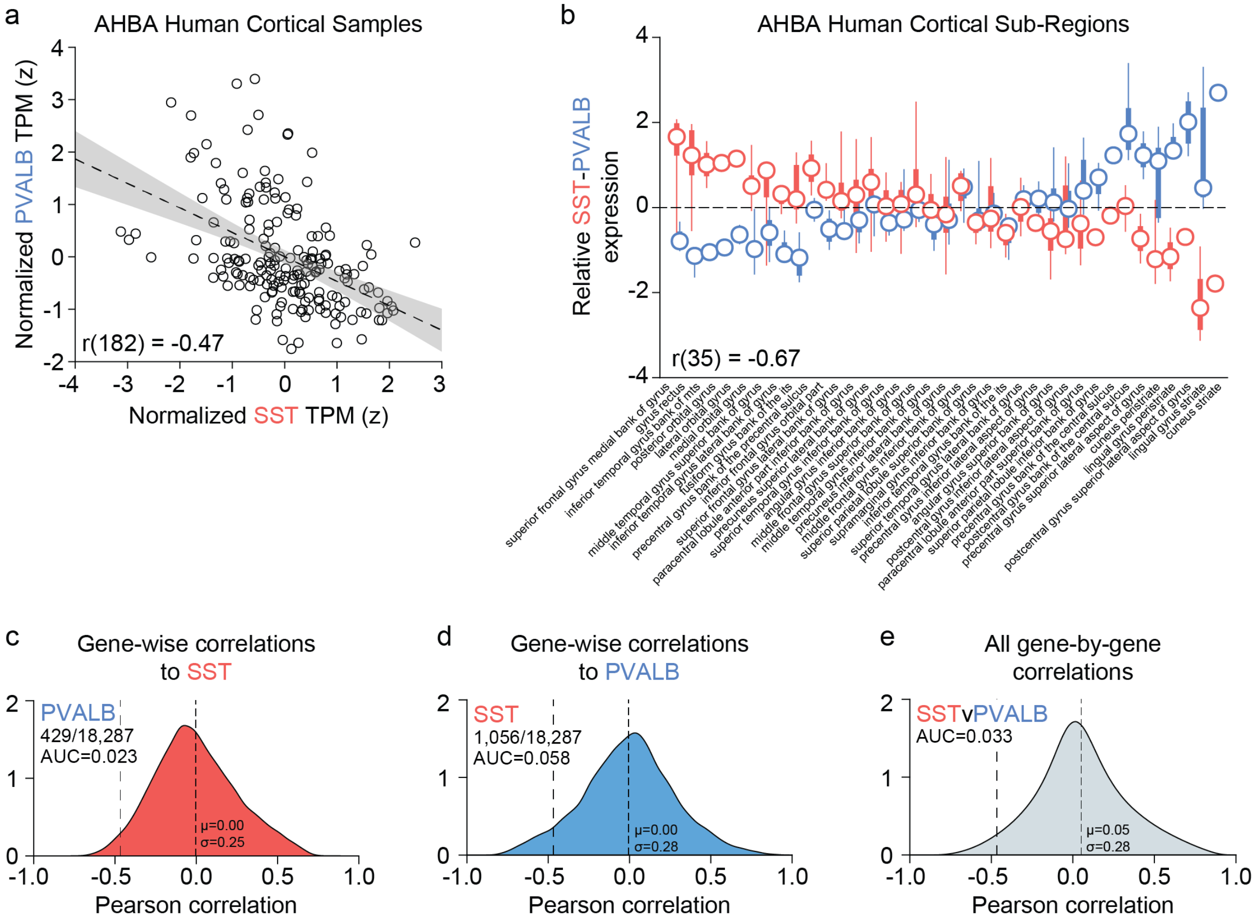

Supplementary Figure 4. Technical replication of SST and PVALB negative relationship. To rule out potential microarray specific artifacts driving the negative SST-PVALB relationship, we replicate our findings using RNA-Seq data from the two bi-hemispheric AHBA donors. (a) Z-transformed *SST* Transcripts Per Million (TPM) was negatively correlated to Z-transformed *PVALB* TPM (*r*(182)=-0.47; p=2.4e-11). (b) The SST-PVALB negative spatial relationship was consistent when data was collapsed by cortical area (*r*(35)=-0.67, p=6.7e-6; n=2 donors, n=184 samples, n=37 regions). (c) Compared to all possible gene-wise correlations, *PVALB* was the 429th most negatively correlated gene to *SST* (AUC=0.023). (d) Compared to all possible gene-wise correlations, *SST* was the 1,056th most negatively correlated gene to *PVALB* (AUC=0.058). (e) Indicating that a negative correlation of *r*=-0.47 is not a common feature of cortex, the SST-PVALB relationship was the 5,464,076 most negative effect relative to 167,216,328 possible gene-wise comparisons (AUC=0.033).

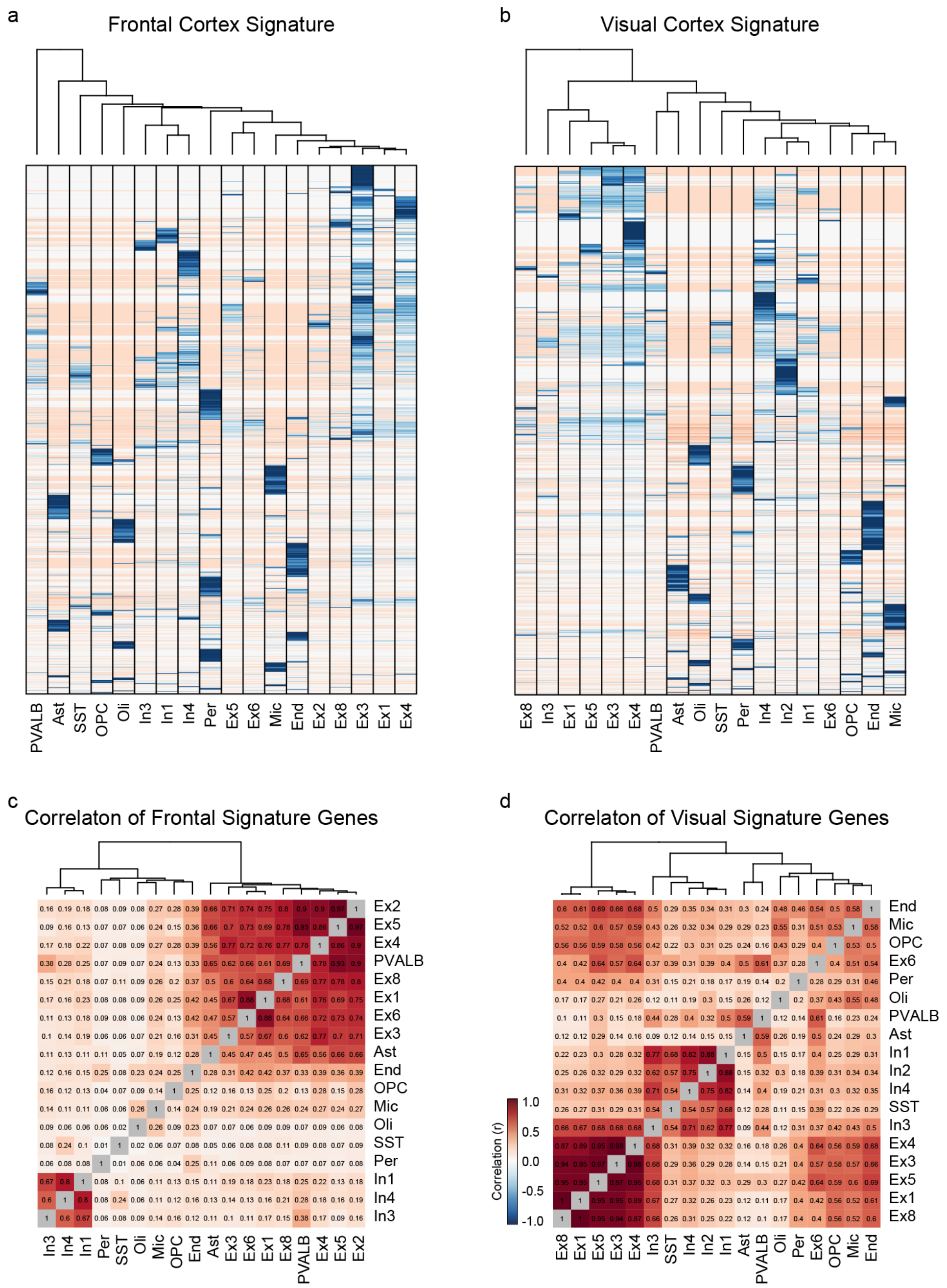

Supplementary Figure 5. Expression profiles of (a) 4,138 signature genes defining cell types from Dorsal Frontal Cortex (DFC; BA6+BA10) and (b) 5,017 genes defining cell types from Visual Cortex (VIS; BA17). Blue indicates greater expression and red indicated reduced expression averaged across each of 18 cell classes. Ast=astrocyte, OPC=oligodendrocyte precurser cells, Oli=oligodendrocyte, Per=pericytes, Mic=microglia, End=endothelial, In=inhibitory, Ex=excitatory. To identify potential collinearity among genetic signatures, average signature gene expression profiles were correlated across (c) frontal and (d) visual cell types, producing an 18x18 matrix where warm red indicated greater transcriptional similarity between cell classes.

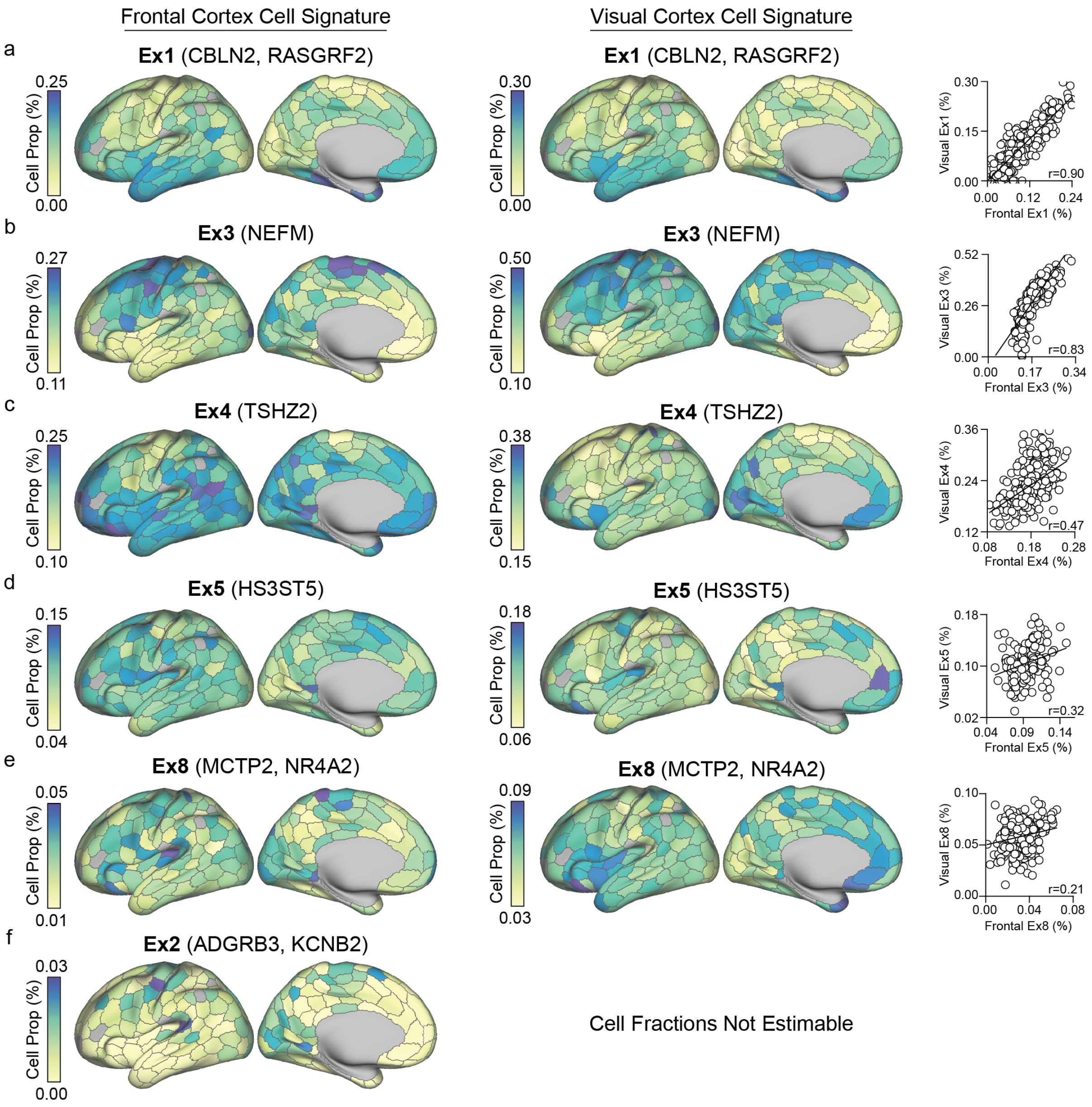

Supplementary Figure 6. Polygenic deconvolution of excitatory neurons across cortex. Spatial distributions of deconvolved cell type fractions in left-hemisphere, averaged in each of 400 parcels from Schaefer and colleagues[1]. Highly stable spatial cell fractions were observed for (a) Ex1 (r=0.90), (b) Ex3 (r=0.83), and (c) Ex4 (r=0.47) cell classes between DFC and VIS based cell signature. Positive, but weaker spatial consistency was observed among (d) Ex5 (r=0.32) and (e) Ex8 (r=0.21) visual cortex and frontal cortex derived spatial maps. (f) Ex2 cells were only estimable using single-cell data from frontal cortex. In parentheses are examples of preferentially expressed genes for each neuron class. Given the polygenic nature of these deconvolutions, however, individual marker genes should be interpreted with caution. Correlation plots display the spatial relationship between frontal and visual signature derived cell fraction maps. That is, each circle in the correlation plot represents an ROI on the cortical surface.

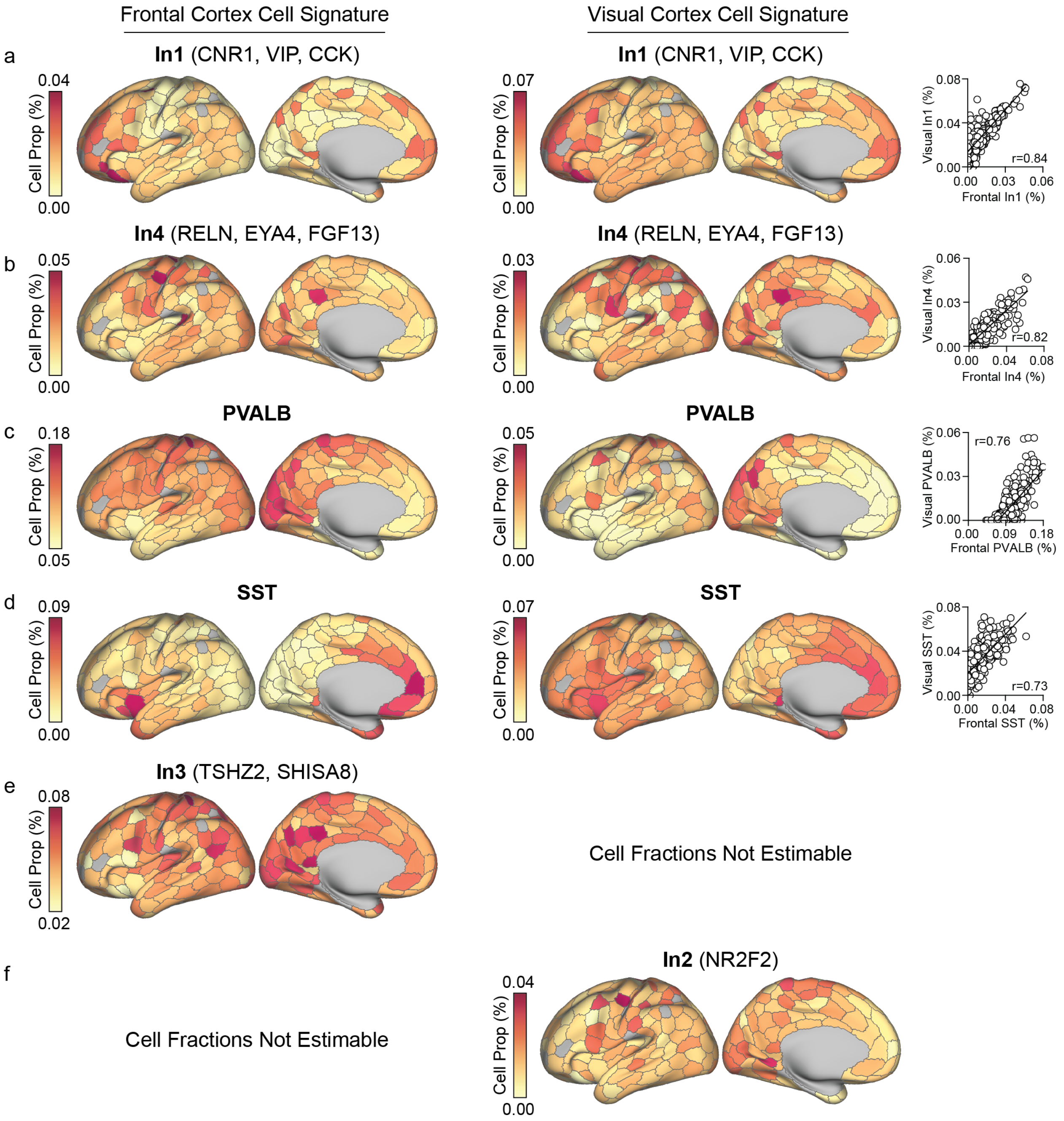

Supplementary Figure 7. Polygenic deconvolution of inhibitory neurons across cortex. Spatial distributions of deconvolved cell type fractions in left-hemisphere (a-f), averaged in each of 400 parcels from Schaefer and colleagues[1]. Correlation plots display the spatial relationship between frontal cortex (DFC) and visual cortex (VIS) signature derived cell fraction maps, which were highly stable for (a) In1 (r=0.84), (b) In4 (r=0.82), (c) PVALB (r=0.76), and SST (r=0.73) cell classes. Each circle in the correlation plots represents an ROI on the cortical surface. Genes in parentheses are example preferentially expressed genes for each neuron class. Given the polygenic nature of these deconvolutions, however, individual marker genes should be interpreted with caution.

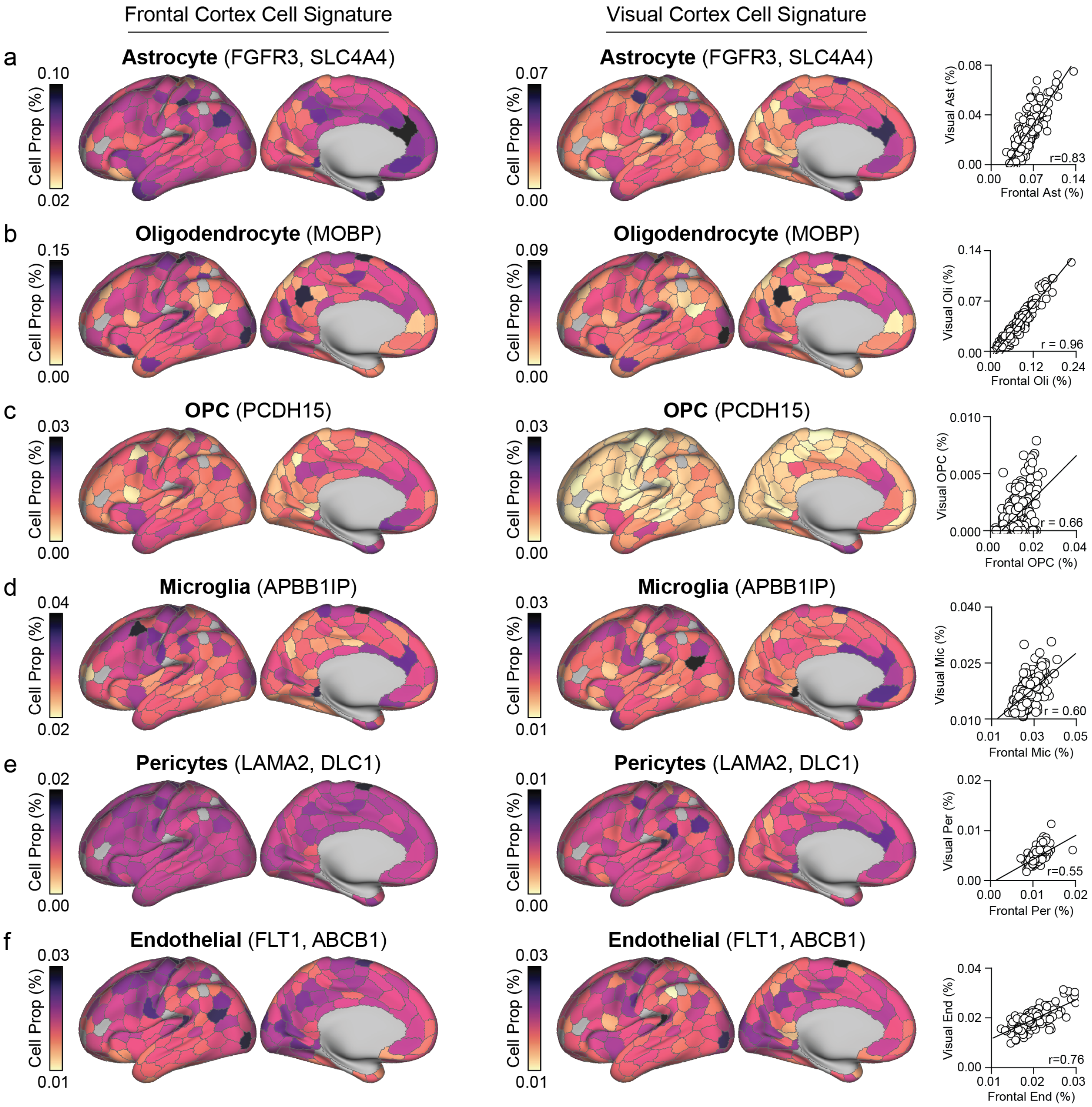

Supplementary Figure 8. Polygenic deconvolution of non-neuronal cells across cortex. Spatial distributions of deconvolved cell type fractions in left-hemisphere (a-f), averaged in each of 400 parcels from Schaefer and colleagues[1]. Correlation plots display the spatial relationship between frontal cortex (DFC) and visual cortex (VIS) signature derived cell fraction maps, which were highly stable for (a) Astrocytes (r=0.83), (b) Oligodendrocytes (r=0.96), (c) OPC (r=0.66), and (d) Microglia (r=0.60), (e) Pericytes (r=0.55), and (f) Endothelial (r=0.76) cell classes. Genes in parentheses are example preferentially expressed genes for each neuron class. Given the polygenic nature of these deconvolutions, however, individual marker genes should be interpreted with caution.

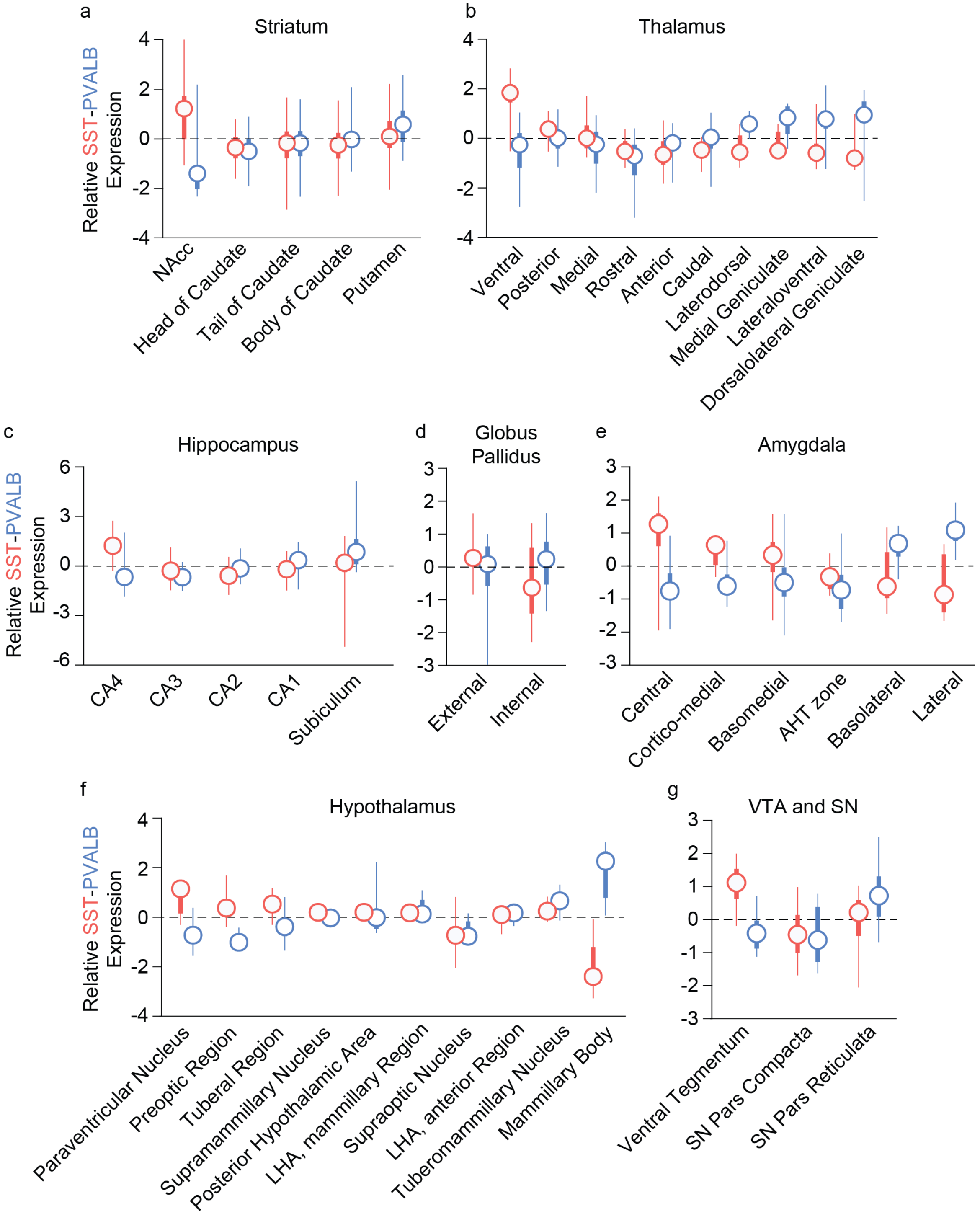

Supplemental Figure 9. Z-transformed expression values of *SST* and *PVALB* for sub-regions of (a) striatum (n=170 samples), (b) thalamus (n=175 samples), (c) hippocampus (n=158 samples), (d) globus pallidus (n=39 samples), (e) amygdala (n=67 samples), (f) hypothalamus (n=102 samples), and (g) combined ventral tegmentum and substantia nigra (n=65 samples). Regions are ordered by relative median expression of *SST* to *PVALB*. Circle=median, thick lines=interquartile range, thin line=minimum and maximum.

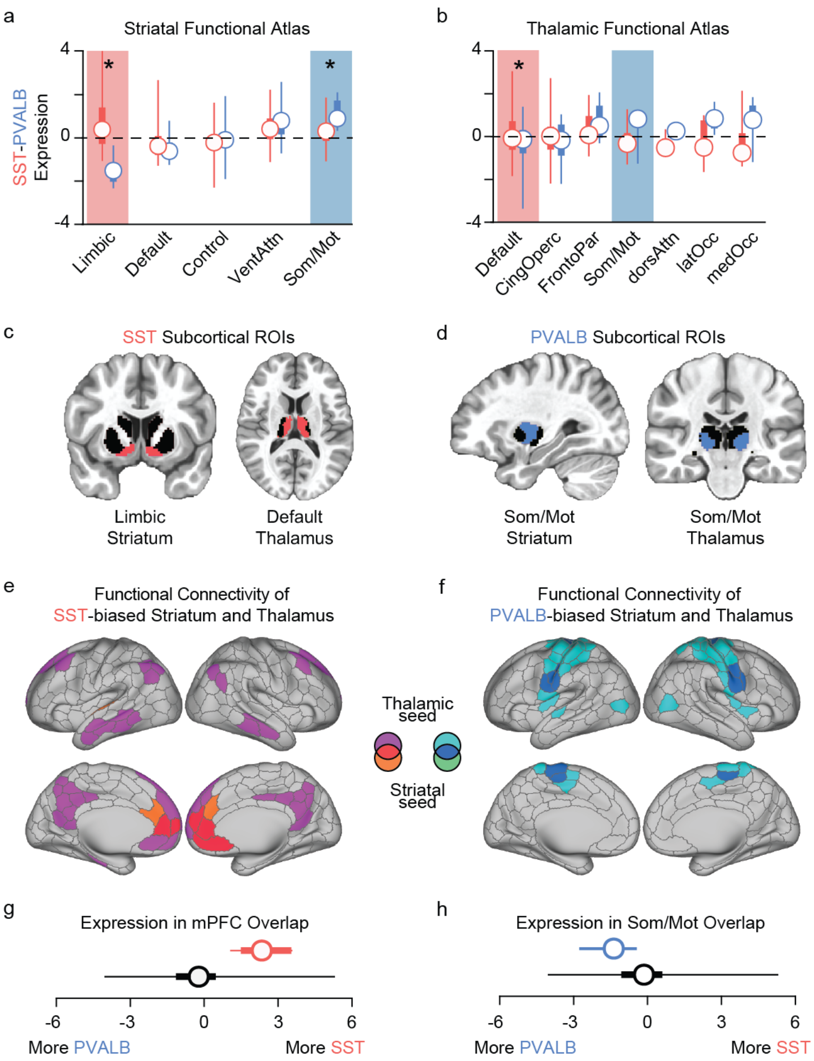

Supplemental Figure 10. Differential *SST* and *PVALB* expression in functional networks of striatum and thalamus. (a and b) Relative *SST* and *PVALB* expression across functionally defined striatal (n=170 samples)[2] and thalamic (n=175 samples)[3] subregions. Normalized *SST* was greater that *PVALB* in Limbic striatum (t(15)=6.57, p=8.8e-6; two-sided; Wilcoxon: W=136, p=3.1e-5), and relative *PVALB* was greater in Som/Mot striatum (t(11)=2.91, p=0.014; two-sided; Wilcoxon: W=11, p=0.03). Normalized *SST* was greater that *PVALB* in Default thalamus (t(73)=2.56, p=0.012; two-sided; Wilcoxon: W=1769, p=0.04), but there was no evidence for greater *PVALB* in Som/Mot thalamus (t(5)=0.99, p=0.37; two-sided; Wilcoxon: W=6, p=0.44). Illustration of striatal and thalamic ROIs of the (c) Limbic and (d) Som/Mot network (e) Limbic striatum and default thalamus possess overlapping positive resting state correlations to *SST*-biased aspects of medial prefrontal cortex (mPFC; *r's*≥0.05). Likewise, (f) somato/motor striatum and thalamus show overlapping positive correlations to *PVALB*-biased portions of somatosensory cortex (r's≥0.03). (g) *SST* expression within the 5 overlapping mPFC limbic parcels is greater than all other cortical parcels. (h) *PVALB* expression within the 14 overlapping somato/motor parcels is greater than all other cortical parcels.

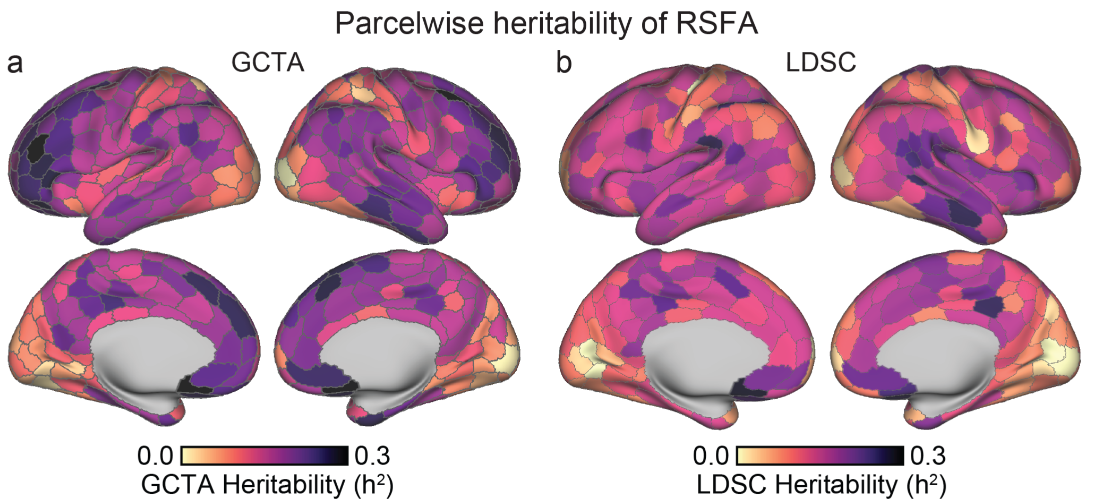

Supplemental Figure 11. SNP heritability for each of the 400 bi-hemispheric cortical parcels from Schaefer and colleagues (n=9,713), calculated using (a) GCTA-GREML and (b) LDSC regression. The two methods produce RSFA heritability estimates that are spatially correlated at r_s=0.61 (p=2.2e-16).

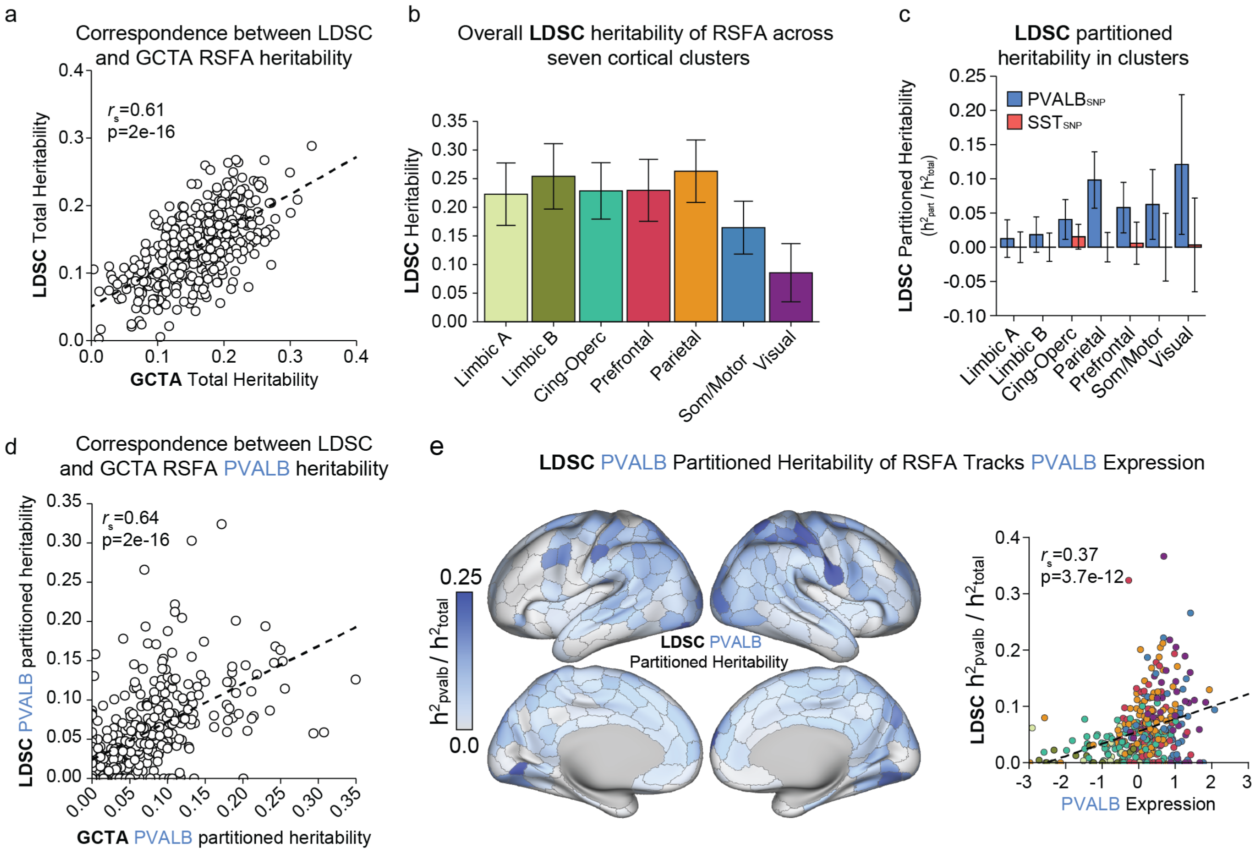

Supplemental Figure 12. Technical replication of GCTA heritability results using LDSC. GCTA derived estimates of heritability and partitioned heritability were highly consistent with results from LDSC. (n=9,713 UKB subjects). (a) Overall heritability of RSFA estimated with GCTA and LDSC were strongly spatially correlated across parcels (r_s=0.61, p=2e-16). (b) LDSC based heritability of RSFA across seven cortical clusters. Error bars represent 95% confidence intervals. (c) LDSC based partitioned heritability of $PVALB_{SNP}$ and SST_{SNP} variant bins. The top 500 genes most spatially correlated to *PVALB* and *SST* in cortex were identified. Higher partitioned heritability values indicate that PVALB and SST account for an enriched proportion of genetic variance in RSFA for a given cluster. Error bars represent 95% confidence intervals. (d) LDSC partitioned heritability of $PVALB_{SNP}$ variants across each cortical parcel was highly spatially consistent with GCTA derived estimates (as shown in Figure 5c, r_s=0.64, p=2e-16). Each circle in the dot plot represents an individual cortical region from the Schaefer 400 parcellation. (e) Spatial plot of LDSC estimates of genetic variance explained by the $PVALB_{SNP}$ set. Darker blue indicated regions where a greater amount of genetic variance is explained by PVALB-linked genes. Providing a technical replication of Figure 5c, PVALB-partitioned heritability was spatially correlated with *PVALB* expression in the AHBA (r_s=0.37, p=3.7e-12).

Supplementary References

1. Schaefer, A. *et al.* Local-global parcellation of the human cerebral cortex from intrinsic functional connectivity MRI. *Cereb. Cortex* **28**, 3095–3114 (2018).
2. Choi, E. Y., Yeo, B. T. T. & Buckner, R. L. The organization of the human striatum estimated by intrinsic functional connectivity. *J. Neurophysiol.* **108**, 2242–2263 (2012).
3. Hwang, K., Bertolero, M. A., Liu, W. B. & D'Esposito, M. The human thalamus is an integrative hub for functional brain networks. *J. Neurosci.* **37**, 5594–5607 (2017).
