# Supplementary material for: Transcriptional and imaging-genetic association of cortical interneurons, brain function, and schizophrenia risk
Source: Nat Commun. 2020 Jun 8;11:2889. doi: 10.1038/s41467-020-16710-x (PMC7280213; doi:10.1038/s41467-020-16710-x)
Supplement: Supplementary file 2 — Description of Additional Supplementary Files [file 41467_2020_16710_MOESM2_ESM.docx]

**Description of Additional Supplementary Files**

**File Name:** Supplementary Data 1

**Description:** Sheets are described individually below.

**1. AHBA_sample_counts**: Allen Human Brain Atlas (AHBA) sample counts for (1) each of 8 overarching anatomical categories, (2) each parcel from the Choi et al. (2012), 7-Network striatal parcellation, and (3) each parcel of the Hwang et al. (2017) thalamic parcellation.

**2. AHBA_sample_ontology:** AHBA sample ontology information for each category of samples analyzed.

**3. SST_cort_cor:** Across all AHBA cortical samples, the spatial correlation of each gene to somatostatin (SST). Pearson correlations were calculated using mean- and variance-normalized expression values.

**4. PVALB_cort_cor:** Across all AHBA cortical samples, the spatial correlation of each gene to parvalbumin (PVALB). Pearson correlations were calculated using mean- and variance-normalized expression values.

**5. LakeDFC_gene_signature:** Gene signature matrix produced by CIBERSORTx, using single-cell data from frontal cortex of Lake and colleagues (2018).

**6. LakeVIS_gene_signature:** Gene signature matrix produced by CIBERSORTx, using single-cell data from visual cortex of Lake and colleagues (2018).

**7. AHBA_cellfrac_DFC:** Estimated fractional abundance of each cell type, using the Lake frontal cortex single-cell data, across all cortical AHBA samples.

**8. AHBA_cellfrac_VIS:** Estimated fractional abundance of each cell type, using the Lake visual cortex single-cell data, across all cortical AHBA samples.

**9. PVALB_SNPs_1_500:** Intragenic (+/- 5000 base pairs from transcription start/stop site) and eQTL SNPs for the 500 genes with the strongest spatial cortical correlation to *PVALB*.

**10. SST_SNPs_1_500:** Intragenic (+/- 5000 base pairs from transcription start/stop site) and eQTL SNPs for the 500 genes with the strongest spatial cortical correlation to *SST*.

**11. SST_top500_enrichment:** ToppGene (https://toppgene.cchmc.org/) ontological enrichment analyses for the 500 genes most correlated to SST. Only genes with genotyped intragenic structural variants in the UK Biobank were considered.

**12. PVALB_top500_enrichment:** ToppGene (https://toppgene.cchmc.org/) ontological enrichment analyses for the 500 genes most correlated to PVALB. Only genes with genotyped intragenic structural variants in the UK Biobank were considered.

**13. RSFA_7Cluster_Stats:** SNP-wise heritability for (1) average resting-state functional amplitude (RSFA) within the 7 data-derived cortical parcels. (2) Partitioned heritability of RSFA within 7 cortical clusters, estimated using intragenic SNPs from the top 500 PVALB-correlated genes, top 500 SST-correlated genes, and all other SNPs. (3) Relationship between parcel-wise RSFA and polygenic risk for schizophrenia.

**14. RSFA_SchaefParcel_Herit:** SNP-wise heritability of RSFA for each of the 400 Schaeffer 7-network cortical ROIs. "Total_h2snp" was calculated using a three-partition GCTA model that included(1) PVALB_snps, (2) SST_snps, and (3) all other SNPs. Also includes normalized SST and PVALB expression within each parcel.

**15. RSFA_SchaefParcel_Herit:** SNP-wise heritability of RSFA for each of the 400 Schaeffer 7-network cortical ROIs. "Total_h2snp" was calculated using a three-partition GCTA model that included(1) PVALB_snps, (2) SST_snps, and (3) all other SNPs.

**16. Brainspan_dev:** Relationship between normalized cortical *SST* and *PVALB* expression, controlling for age and gender, across development groups from the Brainspan Atlas of the Developing Human Brain.

**17. AHBA_Kim_subcortical:** Contains the formatted SST and PVALB cell density estimates from Kim and colleagues (2017). Column “AHBA_acro” contains the human regional name (from AHBA ontology) for matching across datasets.
