# Supplementary material for: Transcriptional and imaging-genetic association of cortical interneurons, brain function, and schizophrenia risk
Source: Nat Commun. 2020 Jun 8;11:2889. doi: 10.1038/s41467-020-16710-x (PMC7280213; doi:10.1038/s41467-020-16710-x)
Supplement: Supplementary file 4 — Reporting Summary [file 41467_2020_16710_MOESM4_ESM.pdf]

nature research

Corresponding author(s): Avram J. Holmes

Last updated by author(s): Mar 4, 2020

Reporting Summary

Nature Research wishes to improve the reproducibility of the work that we publish. This form provides structure for consistency and transparency in reporting. For further information on Nature Research policies, see Authors & Referees and the Editorial Policy Checklist .

Statistics

For all statistical analyses, confirm that the following items are present in the figure legend, table legend, main text, or Methods section.

n/a	Confirmed	
☐	☒	The exact sample size (*n*) for each experimental group/condition, given as a discrete number and unit of measurement
☐	☒	A statement on whether measurements were taken from distinct samples or whether the same sample was measured repeatedly
☐	☒	The statistical test(s) used AND whether they are one- or two-sided<br>*Only common tests should be described solely by name; describe more complex techniques in the Methods section.*
☐	☒	A description of all covariates tested
☐	☒	A description of any assumptions or corrections, such as tests of normality and adjustment for multiple comparisons
☐	☒	A full description of the statistical parameters including central tendency (e.g. means) or other basic estimates (e.g. regression coefficient) AND variation (e.g. standard deviation) or associated estimates of uncertainty (e.g. confidence intervals)
☐	☒	For null hypothesis testing, the test statistic (e.g. *F*, *t*, *r*) with confidence intervals, effect sizes, degrees of freedom and *P* value noted<br>*Give P values as exact values whenever suitable.*
☒	☐	For Bayesian analysis, information on the choice of priors and Markov chain Monte Carlo settings
☒	☐	For hierarchical and complex designs, identification of the appropriate level for tests and full reporting of outcomes
☐	☒	Estimates of effect sizes (e.g. Cohen's *d*, Pearson's *r*), indicating how they were calculated

Our web collection on statistics for biologists contains articles on many of the points above.

Software and code

Policy information about availability of computer code

Data collection

All data analyzed were obtained from publicly available resources (e.g. Allen Human Brain Atlas, UK Biobank).

Data analysis

Custom code for our analyses will be made publicly available at the following URL upon publication:

https://github.com/kevmanderson/2020_NatComm_interneurons_cortical_function_schizophrenia
https://github.com/HolmesLab/2020_NatComm_interneurons_cortical_function_schizophrenia

The following software was used:
PLINK v1.9&v2.0 (http://zzz.bwh.harvard.edu/plink/)
MAGMA v1.07a (https://ctg.cncr.nl/software/magma)
GCTA 1.91.7 (https://cnsgenomics.com/software/gcta/#Download)
PRSice-2 (version 2)(https://choishingwan.github.io/PRSice/)
HCP Workbench v1.3.2 (https://www.humanconnectome.org/software/connectome-workbench)
AFNI version from 2017-08-11 (https://afni.nimh.nih.gov/)
LDSC v1.0.1: https://github.com/bulik/ldsc
CIBERSORTx (no version noted): https://cibersortx.stanford.edu/

resting-state fMRI parcellations:
Schaefer et. al. (2018) cortical parcellation (https://github.com/ThomasYeoLab/CBIG)
Choi et al. (2012) striatal parcellation (https://surfer.nmr.mgh.harvard.edu/fswiki/StriatumParcellation_Choi2012)
Hwang et al. (2017) thalamic parcellation is available upon request to the authors.

For manuscripts utilizing custom algorithms or software that are central to the research but not yet described in published literature, software must be made available to editors/reviewers. We strongly encourage code deposition in a community repository (e.g. GitHub). See the Nature Research guidelines for submitting code & software for further information.

Data

Policy information about availability of data

All manuscripts must include a data availability statement. This statement should provide the following information, where applicable:

- Accession codes, unique identifiers, or web links for publicly available datasets
- A list of figures that have associated raw data
- A description of any restrictions on data availability

1) UK Biobank (https://www.ukbiobank.ac.uk/); Application Number 25163; Researchers must request access.
2) Allen Human Brain Atlas (http://human.brain-map.org/). Freely available for download
3) Macaque cortical expression data from Bernard et. al (https://www.ncbi.nlm.nih.gov/geo/query/acc.cgi?acc=GSE31613). Freely available for download
4) Brainspan Atlas of the Developing Human Brain (http://www.brainspan.org/). Freely available for download
5) NIH GTEx (https://gtexportal.org/home/). Freely available for download
6) CommonMind Consortium (https://www.nimhgenetics.org/available_data/commonmind/). Freely available for download
7) Ripke Schizophrenia GWAS summary stats (https://www.med.unc.edu/pgc/results-and-downloads). Freely available for download
8) Single-cell transcription data from Lake and colleagues (2018; https://www.ncbi.nlm.nih.gov/geo/query/acc.cgi?acc=GSE97930)
9) Rodent cell density estimate from Supplemental Data of Kim and colleagues (2017; https://www.sciencedirect.com/science/article/pii/S0092867417310693)
10) LDSC reference files, freely available here: https://data.broadinstitute.org/alkesgroup
11) ANTs: http://stnava.github.io/ANTs/

Field-specific reporting

Please select the one below that is the best fit for your research. If you are not sure, read the appropriate sections before making your selection.

[x] Life sciences [ ] Behavioural & social sciences [ ] Ecological, evolutionary & environmental sciences

For a reference copy of the document with all sections, see nature.com/documents/nr-reporting-summary-flat.pdf

Life sciences study design

All studies must disclose on these points even when the disclosure is negative.

Sample size

Our analyses utilized open-access genetic and neuroimaging consortia data, so no sample size calculations were performed. Rather, we sought to maximize sample size in all cases, removing samples or subjects with questionable data quality.

UK Biobank imaging analyses (N=9,713). According to the GCTA-GREML calculator available here (https://cnsgenomics.shinyapps.io/gctaPower/), we had 80% power to detect snp-wise heritability > 0 for a phenotype with h2=0.092.

A total of 13,236 UKB subjects were available and able to be processed through the imaging pipeline. Subjects with mean run-wise frame-to-frame head motion greater than 0.20 mm, and inverted resting-state SNR greater than 3 standard deviations above the mean were removed. After filtering for white British subjects with usable genetic data, cryptic relatedness <0.025, and conducting row-wise deletion for the variables age, sex, height, weight, BMI, three head position coordinates (X,Y,Z), combined gray/white matter volume, combined ventricular/CSF volume, diastolic and systolic blood pressure, run-wise resting state motion, resting state inverse SNR, T1 inverse SNR, and UK Biobank assessment center, 9,713 subjects remained for analyses (percent female=54.33, mean age= 63.67 SD= 7.45, min/max age=45-80). We included the anthropometric measures of height, BMI, weight, and blood pressure given previously demonstrated associations with imaging phenotypes in the UK Biobank.

Data exclusions

1) UKBiobank subjects were thresholded based upon SNR (T1 and resting-state) and head motion thresholds, and row-wise deletion was conducted on all analyzed data fields. Last, only genetically unrelated white/non-latino subjects were retained for the final sample (n=9,713). These criteria were not pre-determined, but reflect field standard preprocessing steps.
2) Two macaques in the NIH Blueprint were not excluded due to sparse sampling across the 11 analyzed brain regions (1 primate was sampled in just temporal lobe; 1 primate was only sampled within OFC, DLPFC, and ACC). This exclusion criteria was determined after examining sample counts for each donor in the NIH Blueprint database.

Replication

1) We replicated the SST/PVALB negative cortical correlation observed in AHBA data within macaque NIH Blueprint and human Brainspan data.
2) GCTA-REML results were replicated using stratified LDSC
3) Subcortical SST/PVALB distributions were replicated within rodent data from Kim and colleagues (2018)

No other replications were conducted.

Randomization	Randomization was not relevant to our analyses because they were always conducted on the full set of available data and did not include "case/control" experimental design.
Blinding	Blinding was not relevant since analyses were conducted on publicly available archival data and did not use an experimental/control group design.

Reporting for specific materials, systems and methods

We require information from authors about some types of materials, experimental systems and methods used in many studies. Here, indicate whether each material, system or method listed is relevant to your study. If you are not sure if a list item applies to your research, read the appropriate section before selecting a response.

Materials & experimental systems

n/a	Involved in the study	
☒	☐	Antibodies
☒	☐	Eukaryotic cell lines
☒	☐	Palaeontology
☐	☒	Animals and other organisms
☐	☒	Human research participants
☒	☐	Clinical data

Methods

n/a	Involved in the study	
☒	☐	ChIP-seq
☒	☐	Flow cytometry
☐	☒	MRI-based neuroimaging

Animals and other organisms

Policy information about studies involving animals; ARRIVE guidelines recommended for reporting animal research

Laboratory animals	Macaca mulatta (2 Male; 2 Female). Specific age information was not available, however the animals were adults and Bernard et al (2012) reported their age as (M=8.5, SE=1.0 years).
Wild animals	The study did not involve wild animals
Field-collected samples	The study did not involve field-collected samples
Ethics oversight	Analyses were conducted according to the guidelines of the Yale University IRB

Note that full information on the approval of the study protocol must also be provided in the manuscript.

Human research participants

Policy information about studies involving human research participants

Population characteristics	UK Biobank imaging genetic sample (N=9,713; female: 54.33; Age: 63.67 (SD 7.45), min=45, max=80; All White/Non-Latino)
Recruitment	Information about UK Biobank recruitment is published. Miller, K. L. et al. Multimodal population brain imaging in the UK Biobank prospective epidemiological study. Nat. Neurosci. 19, 1523–1536 (2016).
Ethics oversight	Analyses were conducted according to the guidelines of the Yale University Human Subjects Committee

Note that full information on the approval of the study protocol must also be provided in the manuscript.

Magnetic resonance imaging

Experimental design

Design type	resting state functional magnetic resonance imaging
Design specifications	One 6-minute rest-run per subject (490 timepoints; TR=0.735)
Behavioral performance measures	We analyzed resting-state imaging data, which does not have an associated behavioral metric.

Acquisition

Imaging type(s): resting-state functional MRI

Field strength: 3

Sequence & imaging parameters: Resolution: 2.4x2.4x2.4 mm; Field-of-view: 88x88x64 matrix; Duration: 6 minutes (490 timepoints); TR: 0.735 s; TE: 39ms; GE-EPI with x8 multislice acceleration, no iPAT, flip angle 52∘, fat saturation

Area of acquisition: whole-brain

Diffusion MRI: ☐ Used ☒ Not used

Preprocessing

Preprocessing software: Software:
AFNI (binaries downloaded on 2017-08-11)
HCP Workbench v1.3.2 (https://www.humanconnectome.org/software/connectome-workbench)

Normalization: Linear and non-linear transformations were calculated by the UK Biobank. T1 images were nonlinearly transformed to MNI152 "nonlinear 6th generation" standard-space using FNIRT.
EPI unwarping occurred in a combined step that included linear alignment to T1 using FLIRT (BBR cost function), that accounted for shifts resulting from GDC unwarping. The nonlinear transformation to MNI space (described above) is then applied.

Normalization template: MNI152 "nonlinear 6th generation"

Noise and artifact removal: Noise reduction steps included (1) ICA+FIX removal of structured artifacts, (2) 3dDespike, (3) regression of CSF and WM signal, (4) first and second order trend removal, and (5) fast ANATICOR to remove local WM signal (ANATICOR conducted for resting-state connectivity analysis only).
Subjects with averaged frame-to-frame head motion greater than 0.2mm were excluded, as were subjects with inverse T1 SNR or inverse rest SNR greater than 3 standard deviations from the group mean.

Volume censoring: Volumes were not censored

Statistical modeling & inference

Model type and settings: We did not conduct model based analyses of neuroimaging data.

Effect(s) tested: We tested whether resting-state functional amplitude was correlated to post-mortem expression of interneuron gene markers

Specify type of analysis: ☐ Whole brain ☒ ROI-based ☐ Both

Anatomical location(s): ROIs were defined using the Schaefer cortical, Choi striatal, and Hwang thalamic functional atlases.
Schaefer, A. et al. Local-global parcellation of the human cerebral cortex from intrinsic functional connectivity MRI. Cereb. Cortex 28, 3095–3114 (2018).
Choi, E. Y., Yeo, B. T. T. & Buckner, R. L. The organization of the human striatum estimated by intrinsic functional connectivity. J. Neurophysiol. 108, 2242–2263 (2012).
Hwang, K., Bertolero, M. A., Liu, W. B. & D'Esposito, M. The human thalamus Is an integrative hub for functional brain networks. J. Neurosci. 37, 5594–5607 (2017).

Statistic type for inference (See Eklund et al. 2016): Analyses were conducted at the cluster and individual parcel level.

Correction: Where relevant, statistical tests were corrected for multiple comparisons.

Models & analysis

n/a	Involved in the study
☐	☒ Functional and/or effective connectivity
☒	☐ Graph analysis
☒	☐ Multivariate modeling or predictive analysis

Functional and/or effective connectivity: Z-transformed (within-subject) pearson correlations between each of 400 roughly symmetric cortical ROIs from the parcellation of Schaefer et al. (2018)
